# Box-Behnken optimized copper oxide nanoparticles from *Thymus vulgaris* potentiate efficacy against multidrug-resistant bacterial pathogens and exhibit anticancer activity

**DOI:** 10.1186/s40643-026-01008-5

**Published:** 2026-02-11

**Authors:** Samah H. Abu-Hussien, Akebe Luther King, Muhammad A. Khan

**Affiliations:** 1 Department of Agricultural Microbiology, Faculty of Agriculture, Ain Shams University, Cairo, 11241 Egypt; 2 Total Environment Research (TEN-R) Group, College of Health Sciences, University of KwaZulu-Natal, Berea, South Africa; 3 Environmental Research Foundation, Westville, South Africa; 4 Department of Biological Sciences, Faculty of Sciences, International Islamic University (IIU), Islamabad, Pakistan

**Keywords:** Box-Behnken optimization, Copper oxide nanoparticles, Antimicrobial synergy, Antibiotic resistance, Green nanotechnology

## Abstract

**Abstract:**

The dual crises of antimicrobial resistance and cancer demand innovative therapeutic platforms that overcome conventional treatment limitations. This study uniquely combines systematic Box-Behnken optimization of green-synthesized copper oxide nanoparticles from *Thymus vulgaris* with comprehensive evaluation of their synergistic antimicrobial and anticancer activities. HPLC profiling identified quercetin (55.92%), chlorogenic acid (15.33%), and gallic acid (12.28%) as principal phytochemical reducing and capping agents. Statistical optimization (R^2^ = 0.9886) established copper acetate concentration (F = 670.48, *p* < 0.0001) and incubation time (F = 124.11, *p* < 0.0001) as critical synthesis determinants, yielding monodisperse spherical nanoparticles (19–25 nm TEM; Z-average 119.2 nm, PDI 0.22; ζ-potential − 45.8 mV). XRD confirmed a crystalline monoclinic CuO phase, while FTIR validated phytochemical surface functionalization. TE-CuONPs exhibited concentration-dependent bactericidal activity (MIC 250–950 μg/mL; MBC/MIC ≤ 0.58) against *Staphylococcus aureus*, *Pseudomonas aeruginosa*, *Escherichia coli*, and *Enterococcus faecalis* as well as inhibition of biofilm formation in *S. aureus* and *P. aeruginosa*, with BIC₅₀ of 299 and 315 μg/mL, respectively. Critically, checkerboard assays revealed strong synergy with gentamicin (FICI 0.13–0.28), achieving eightfold dose reduction for both agents against *S. aureus* and *P. aeruginosa*. Time-kill kinetics demonstrated accelerated bacterial eradication, with combination therapy achieving ≥ 3-log₁₀ reduction 8–12 h faster than monotherapies, a clinically significant advantage for acute infections. Furthermore, TE-CuONPs showed moderate antiproliferative activity (IC₅₀ = 117.26 μg/mL) against MCF-7 breast cancer cells, with limited selectivity over normal fibroblasts (SI = 1.85), representing a sixfold enhancement over the crude extract. Additionally, Flow cytometric analysis revealed profound apoptotic induction, with 77.25% of cancer cells undergoing cell death (29.73% early apoptosis, 47.52% late apoptosis/necrosis). DPPH radical scavenging (IC₅₀ = 55 μg/mL) demonstrated a threefold superior antioxidant capacity versus plant extract alone. These findings advance the reproducible botanical nanoparticle synthesis and translational potential of plant-mediated nanomedicine for infectious disease management.

**Graphical abstract:**

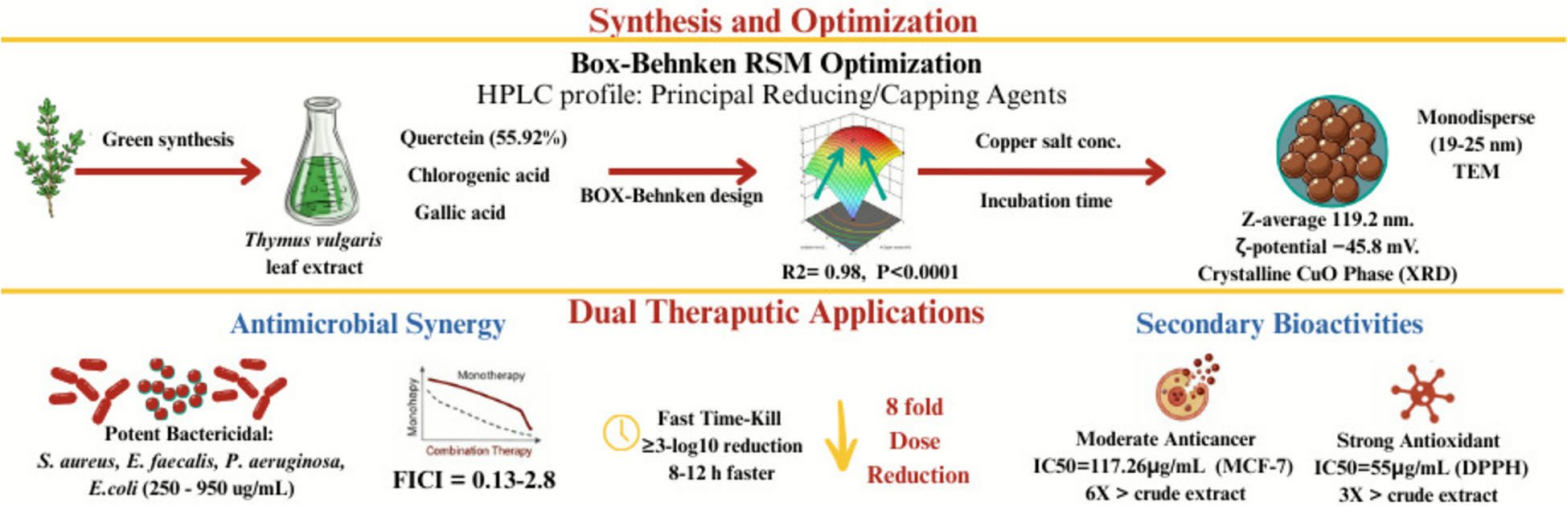

**Supplementary Information:**

The online version contains supplementary material available at 10.1186/s40643-026-01008-5.

## Introduction

Antimicrobial resistance represents one of the most urgent global health challenges, with multidrug-resistant bacterial infections causing approximately 1.27 million deaths annually and projected economic losses exceeding $100 trillion by 2050 if current trends continue (Church and McKillip 2021 ). Conventional antibiotic monotherapies are increasingly compromised by resistance mechanisms, including enzymatic degradation, target site modifications, and enhanced efflux pump expression (Munita and Arias 2016; Bougnom et al. 2019; El-Zanfaly et al. 1987; Martínez and Baquero 2014; Walsh 2000; Azzam et al. 2017; Abia et al. 2015; Noor et al. 2021; Abia and Essack 2023; Ahmed et al. 2020). Combination therapy strategies that employ complementary mechanisms of action offer a promising approach to overcome resistance, reduce therapeutic doses, and minimize selective pressure for resistance development (Sung et al. 2021; Mohanpuria et al. 2008).

Metallic nanoparticles have emerged as potential antimicrobial adjuvants due to their multi-targeted mechanisms that circumvent single-gene resistance pathways (Dikshit et al. 2021). Among these, copper oxide nanoparticles (CuONPs) demonstrate broad-spectrum bactericidal activity through reactive oxygen species (ROS) generation, membrane disruption, and intracellular ion release (Tiwari et al. 2016; Ramos-Zúñiga et al. 2023). However, clinical translation remains hindered by synthesis irreproducibility, batch-to-batch variability, and inadequate systematic optimization (Asamoah et al. 2020). Green synthesis using plant extracts offers an environmentally sustainable alternative to chemical methods, with phytochemicals serving dual roles as reducing agents and stabilizing ligands (Makabenta et al. 2021; Kumar et al. 2019). *Thymus vulgaris* L., rich in polyphenolic compounds including rosmarinic acid, quercetin, and thymol, represents a particularly promising candidate due to its high reducing capacity and intrinsic antimicrobial properties (Miu and Dinischiotu 2022; Salem and Fouda 2021).

The importance of green synthesis of nanoparticles lies in its eco-friendly and sustainable approach, which leverages plant metabolites as natural reducing and stabilizing agents, overcoming the toxicity and environmental hazards of conventional chemical methods (El-Zmrany et al. 2025). Studies have demonstrated that phytochemicals such as polyphenols, flavonoids, and organic acids play crucial roles in reducing metal ions to nanoparticles and capping them to enhance stability and biological activity (Abu-Hussien et al. 2025; Selim et al. 2025). These natural metabolites facilitate controlled nucleation and growth, influencing the size, morphology, and functional properties of nanoparticles, which in turn improve their efficacy in biomedical applications including antimicrobial and anticancer therapies (Alruhaili et al. 2025). This biosynthesis approach aligns with increasing demands for green nanotechnology and offers a promising route for scalable and reproducible nanoparticle production with reduced environmental footprint, as supported by recent literature in sustainable nanoparticle fabrication and drug delivery systems (Gholami et al. 2018; Shakib et al. 2022, 2023; Marzban et al. 2022). Nanomaterials have emerged as a transformative platform in modern biomedical applications due to their tunable physicochemical properties, high surface area, and biocompatibility. Beyond their well-established antimicrobial and environmental uses, nanomaterials are increasingly explored in cancer therapy, offering remarkable potential for targeted drug delivery, photothermal and photodynamic therapies, and immune system modulation. Their nanoscale dimensions allow preferential accumulation at tumor sites through the enhanced permeability and retention (EPR) effect, while surface functionalization enables selective interaction with cancer cells, minimizing systemic toxicity. Moreover, metallic and metal oxide nanoparticles can generate reactive oxygen species under light irradiation, triggering localized cell death and enhancing therapeutic efficacy. These versatile properties highlight nanomaterials as promising candidates for integrated cancer management strategies, combining precision targeting with immunoactivation and minimal side effects (Dosoky et al. 2015; Aruguete et al. 2013; Yonathan et al. 2022; Maxwell et al. 2021; Bradford et al. 2009; El-Liethy et al. 2023).

However, three critical gaps persist: lack of systematic optimization using response surface methodology; limited synergy evaluation for green-synthesized CuONPs against resistant pathogens and poor understanding of synthesis-property-activity relationships. This study aimed to optimize the green synthesis of copper oxide nanoparticles (TE-CuONPs) using *Thymus vulgaris* extract and evaluate their synergistic antimicrobial, anticancer, and antioxidant activities, highlighting their potential as a dual-action nanoplatform for infection control and cancer therapy.

## Materials and methods

### Chemicals used

All materials and reagents used were of analytical or cell culture grade to ensure experimental consistency and reproducibility. Copper(II) acetate monohydrate and sodium hydroxide (NaOH) were purchased from Sigma-Aldrich with analytical purity. Culture media included Mueller–Hinton broth (Oxoid, CM0405) and Mueller–Hinton agar (Oxoid, CM0337), alongside Tryptone Glucose Yeast Extract Agar (Sigma-Aldrich). Antibiotic disks, gentamicin (10 µg, Oxoid CT0425), tetracycline (30 µg, Oxoid CT0051), ampicillin (10 µg, Oxoid CT0016), and norfloxacin (10 µg, Oxoid CT0059), were sourced from Oxoid. Cell culture reagents such as RPMI-1640 medium and fetal bovine serum (FBS) were obtained from Gibco (Thermo Fisher), supplemented with penicillin–streptomycin solution from the same brand. Additional reagents included 2,2-diphenyl-1-picrylhydrazyl (DPPH), MTT, and dimethyl sulfoxide (DMSO) from Sigma-Aldrich, as well as flow cytometry dyes Annexin V-FITC (BD Biosciences) and propidium iodide (BD Pharmingen). All reagents were used according to manufacturer specifications to maintain quality and reliability of results.

### Collection of *Thymus vulgaris* leaves

*T. vulgaris* dried leaves were obtained from the National Research Centre (NRC), Cairo, Egypt, and taxonomically authenticated by NRC experts. The material represents standard commercial-grade thyme commonly used in phytochemical research. The plant material was thoroughly washed, shade-dried, and finely powdered before extraction. A mixture of 200 g of plant powder and 1000 mL of water was heated to 90°C in a round-bottom flask, filtered to remove debris, and stored at 4 ± 1 °C in dark conditions to maintain phytochemical stability. The extract was used directly, without further purification, for the green synthesis of copper oxide nanoparticles (TE-CuONPs) and subsequent biological evaluations (Mohamed et al. 2025).

### Microorganisms and culture media

*Pseudomonas aeruginosa*, *Escherichia coli*, *Enterococcus faecalis*, and *Staphylococcus aureus* were obtained from the Microbial Resources Center (MIRCEN), Cairo, Egypt, and their identities were verified by 16S rRNA sequencing and deposited in GenBank with gene accession numbers PX505286, PX505285, PX457002 and PX457001, respectively. Bacterial strains were maintained on Tryptone Glucose Yeast Extract Agar (TGYA), (Sigma-Aldrich, USA). For long-term preservation and to ensure genetic stability, cultures were stored at 4 °C and routinely subcultured prior to experimental use (Abu-Hussien et al. 2025a). Before use, these bacteria were cultured overnight in TGY broth (30 °C, 120 rpm, Lab-line Ltd. rotary shaker) to obtain a standardized inoculum of 7.0 × 10^5^ viable cells/mL for experimental applications (Abu-Hussien et al. 2025a).

### Identification and quantification of phenolic and flavonoid compounds by HPLC

The phytochemical profile of phenolic acids and flavonoids was determined using reverse-phase high-performance liquid chromatography (RP-HPLC) coupled with ultraviolet–visible (UV–Vis) detection. The analysis was performed on a C18 stationary phase column under a binary gradient elution system consisting of mobile phase A (ultrapure water containing 0.1% formic acid) and mobile phase B (acetonitrile containing 0.1% formic acid). Before injection, samples were filtered through a 0.22 μm membrane filter and then introduced into the chromatographic system. Detection was carried out at a wavelength of 280 nm. Individual phytochemicals were identified by comparing their retention times with those of authentic standards, while quantification was performed using the external standard method based on calibration curves constructed from peak areas versus known concentrations (Khan et al. 2025).

### Synthesis of copper oxide nanoparticles from *T. vulgaris* leaves extract

Copper oxide nanoparticles (TE-CuONPs) were synthesized via a green co-precipitation method using *T. vulgaris* leaf extract as a natural reducing and stabilizing agent. Briefly, 1 g of copper(II) acetate monohydrate was added to 100 mL of freshly prepared *T. vulgaris* extract under continuous magnetic stirring at 50 °C for 2 h. The pH was adjusted to 8 by the dropwise addition of 0.1 M NaOH solution while stirring. The appearance of a dark brown precipitate confirmed nanoparticle formation. The precipitate was collected by centrifugation at 10,000 rpm for 15 min, washed three times with distilled water to remove impurities, and dried in a hot-air oven at 80 °C for 48 h. Finally, the dried material was calcined at 200 °C for 2 h. The phytochemicals in the *T. vulgaris* extract acted as bio-reductants and capping agents, enabling a sustainable, eco-friendly, and non-toxic synthesis of CuONPs (Asemani and Anarjan 2019).

### Optimization of TE-CuONPs synthesis using box-behnken design

The green synthesis of copper oxide nanoparticles (TE-CuONPs) was systematically optimized using a Box-Behnken design (BBD) (Table 1) integrated with response surface methodology (RSM) (Design-Expert® v11.0.3.0, Stat-Ease Inc., USA). Four synthesis parameters were investigated, each at three levels—high (+ 1), medium (0), and low (− 1) -as follows: copper acetate concentration (Factor A, 2–6 mM), pH (Factor B, 7–9), temperature (Factor C, 60–80 °C), and incubation time (Factor D, 4–12 h). The experimental matrix comprised 29 runs, including six center-point replicates to evaluate model reproducibility and pure error. The *T. vulgaris* aqueous leaf extract served as both a reducing and capping agent, with its phytochemical constituents mediating copper ion reduction through chelation and electron-transfer mechanisms (El-Naga et al. 2025; Alsenosy et al. 2024). Two response variables were evaluated: (i) nanoparticle yield (%) calculated as:

$$ {\mathrm{Yield}}\left( \% \right) = \left( {{\mathrm{W}}_{{\mathrm{f}}} {\mathrm{/W}}_{{\mathrm{i}}} } \right) \times {1}00 $$

where W_f represents the mass of purified, calcined CuO nanoparticles and W_i denotes the initial copper precursor mass; and (ii) UV–visible absorbance intensity (a.u.) measured at the characteristic wavelength. Experimental data were fitted to a second-order polynomial regression model:

$$ {\mathrm{Y}} = \beta_{i} + \, \Sigma \beta_{i} {\mathrm{x}}_{i} + \Sigma \beta_{ii} {\mathrm{x}}_{i} + \Sigma \beta_{ij} {\mathrm{x}}_{i} {\mathrm{x}}_{j} $$

where Y is the predicted response, β₀ is the intercept, β_i_, β_i__i_, and β_i__j_ represent linear, quadratic, and interaction coefficients, respectively, and x_i_ and x_j_ denote coded independent variables. Model adequacy was assessed through analysis of variance (ANOVA), coefficient of determination (R^2^), adjusted R^2^, predicted R^2^, adequate precision, and lack-of-fit tests. Three-dimensional response surface and contour plots were generated to visualize factor interactions and identify optimal synthesis conditions (Haji et al. 2022; Vasiliev et al. 2023).

**Table 1:** Box–Behnken design matrix with experimental factors for CuO nanoparticle synthesized using *T. vulgaris* extract

Run No	Factor A: Copper acetate (mM)	Factor B: pH	Factor C: Temperature (°C)	Factor D: Incubation time (h)
1	4 (0)	8 (0)	80 (+ 1)	8 (0)
2	4 (0)	8 (0)	70 (0)	8 (0)
3	6 (+ 1)	7 (− 1)	70 (0)	8 (0)
4	4 (0)	8 (0)	70 (0)	8 (0)
5	4 (0)	7 (− 1)	70 (0)	12 (+ 1)
6	4 (0)	8 (0)	70 (0)	8 (0)
7	4 (0)	7 (− 1)	60 (− 1)	8 (0)
8	2 (− 1)	8 (0)	70 (0)	12 (+ 1)
9	4 (0)	8 (0)	70 (0)	8 (0)
10	4 (0)	8 (0)	80 (+ 1)	12
11	4 (0)	7 (− 1)	70 (0)	4 (− 1)
12	4 (0)	9 (+ 1)	60 (− 1)	8 (0)
13	2 (− 1)	7 (− 1)	70 (0)	8 (0)
14	4 (0)	8 (0)	60 (− 1)	4 (− 1)
15	6 (+ 1)	8 (0)	60 (− 1)	8 (0)
16	4 (0)	8 (0)	60 (− 1)	12 (+ 1)
17	4 (0)	9 (+ 1)	80 (+ 1)	8 (0)
18	2 (− 1)	8 (0)	60 (− 1)	8 (0)
19	2 (− 1)	8 (0)	80 (+ 1)	8 (0)
20	4 (0)	8 (0)	70 (0)	8 (0)
21	2 (− 1)	9 (+ 1)	70 (0)	8 (0)
22	2 (− 1)	8 (0)	70 (0)	4 (− 1)
23	4 (0)	7 (− 1)	80 (+ 1)	8 (0)
24	4 (0)	9 (+ 1)	70 (0)	4 (− 1)
25	2 (− 1)	8 (0)	70 (0)	8 (0)
26	6 (+ 1)	8 (0)	80 (+ 1)	8 (0)
27	4 (0)	8 (0)	70 (0)	8 (0)
28	6 (+ 1)	8 (0)	70 (0)	12 (+ 1)
29	6 (+ 1)	8 (0)	70 (0)	4 (− 1)

### Characterization of TE-CuONPs

Following synthesis, the physicochemical characteristics of the biosynthesized TE-CuONPs were analyzed using a suite of advanced analytical techniques. Ultraviolet–visible (UV–Vis) spectroscopy (UV Analyst-CT 8200) was employed to examine the optical absorption profile of the nanoparticles within the range of 200–800 nm. Fourier-transform infrared spectroscopy (FTIR; Shimadzu Tracer-100) was performed over the spectral range of 1000–4000 cm^−1^ at a resolution of 4 cm^−1^ to confirm the participation of *T. vulgaris* phytoconstituents in nanoparticle capping and stabilization. The hydrodynamic particle size distribution and polydispersity index (PDI) were determined using dynamic light scattering (DLS) with a Nano ZS analyzer (Malvern Instruments, UK). High-resolution transmission electron microscopy (HR-TEM; JEOL JEM-2100, 200 kV) provided detailed insights into nanoparticle morphology and internal structure. Field emission scanning electron microscopy (FE-SEM; JEOL JSM-7800F, 15 kV) was utilized to assess surface topology and elemental composition, while X-ray diffraction (XRD; Bruker D2 Phaser) with Cu Kα radiation (λ = 1.5406 Å) was used to confirm the crystalline structure and phase purity. The average crystallite size was calculated from XRD data using the Debye–Scherrer equation:

$$ {\mathrm{D}} = {\mathrm{k}}\lambda /\left( {\beta {\mathrm{cos}}\theta } \right) $$

where *D* is the mean crystallite size, *λ* is the X-ray wavelength, *β* is the full width at half maximum (FWHM) of the diffraction peak in radians, *θ* is the Bragg angle, and *k* is the shape factor (commonly 0.9) (Ali et al. 2023).

### Antimicrobial activity assessment

#### Antibiotic susceptibility test

Antimicrobial susceptibility was assessed using the Kirby-Bauer method following CLSI guidelines (M02-A13, 2023). Log-phase bacterial cultures grown in Mueller–Hinton broth (Oxoid, UK) were standardized to 0.5 McFarland turbidity (≈1.5 × 10⁸ CFU/mL). Aliquots (100 μL) were spread onto Mueller–Hinton agar plates, and antibiotic disks of gentamicin (10 μg), tetracycline (30 μg), ampicillin (10 μg), and norfloxacin (10 μg) (Oxoid, UK) were applied aseptically. After incubation at 37 °C for 18–24 h, inhibition zones were measured and interpreted according to the CLSI breakpoint criteria (M100-Ed33, 2023). Multidrug resistance (MDR) was defined as resistance to ≥ 1 agent in ≥ 3 antimicrobial categories. All assays were performed in triplicate with technical duplicates.

#### Agar well diffusion assay

The antimicrobial activity of TE-CuONPs was assessed against methicillin-resistant *S. aureus*, *P. aeruginosa*, *E. coli*, and *E. faecalis* using the agar well diffusion method on Mueller–Hinton agar (MHA; Oxoid, UK). Microbial suspensions adjusted to 0.5 McFarland turbidity (≈1.5 × 10⁸ CFU/mL) were diluted 1:100, and 100 μL aliquots were evenly spread onto agar plates using sterile swabs. Wells (6 mm diameter) were aseptically bored, and 100 μL of filter-sterilized (0.22 μm) TE-CuONP suspensions (25–400 μg/mL or respective controls were added. Gentamicin (10 μg/mL) served as the positive control, while sterile distilled water was used as the negative control. Plates were incubated at 37 °C for 24 h, after which inhibition zone diameters (IZD, mm) were measured using a ruler (Bankier et al. 2019). Quality control strains -*E. coli* ATCC 25922, *S. aureus* ATCC 25923, and *P. aeruginosa* ATCC 27853—were included in each experimental run to ensure test reliability. The gentamicin disk diffusion zones for these QC strains measured 19–26 mm for *E. coli*, 19–27 mm for *S. aureus*, and 16–21 mm for *P. aeruginosa*, all falling within the CLSI-recommended acceptable ranges. The inoculum density was confirmed through viable plate counts, corresponding to 1.2–1.8 × 10⁸ CFU/mL, equivalent to the 0.5 McFarland standard (Alsaraf et al. 2020).

### Minimum inhibitory and bactericidal concentrations

Minimum inhibitory concentration (MIC) and minimum bactericidal concentration (MBC) were determined according to CLSI guidelines (M07-A11) using the broth microdilution method in 96-well plates. TE-CuONP stock suspensions (4000 μg/mL in sterile water) and gentamycin (1 mg/mL) were serially two-fold diluted in Mueller–Hinton broth. Each well received 100 μL of the diluted antimicrobial agent and 100 μL of standardized bacterial inoculum (0.5 McFarland, approximately 1.5 × 10⁸ CFU/mL) were diluted 1:100 in sterile saline. Subsequently, 100 μL of the diluted suspension was added to 100 μL of the test medium in each well, resulting in a final bacterial concentration of 7.5 × 10^5^ CFU/mL. Plates were incubated at 37 °C for 24 h, and optical density at 600 nm (OD₆₀₀) was measured using a microplate reader (BioTek Epoch, USA). The MIC was defined as the lowest concentration showing no visible growth (OD₆₀₀ ≤ mean of negative control + 2SD). For MBC determination, 10 μL from wells exhibiting complete inhibition were subcultured onto Mueller–Hinton agar and incubated at 37 °C for 24 h. The MBC was recorded as the lowest concentration resulting in ≥ 99.9% reduction in viable cells compared with the initial inoculum (Alsaraf et al. 2020).

### Time-kill kinetics

Time-kill assays were conducted following CLSI M26-A guidelines with slight modifications. Bacterial cultures (1.0 × 10⁶ CFU/mL, logarithmic phase) were treated with TE-CuONPs (at MBC), *T. vulgaris* extract alone (at equivalent phytochemical concentration), reference antibiotics (at MBC), or left untreated as growth controls in sterile conical flasks containing 20 mL of Mueller–Hinton broth. Cultures were incubated at 37 °C with continuous orbital shaking (150 rpm). At 0, 2, 4, 6, 8, and 24 h, 100 μL aliquots were aseptically withdrawn, serially diluted tenfold in sterile phosphate-buffered saline (PBS, pH 7.4), and 100 μL was plated in duplicate on Mueller–Hinton agar. After incubation at 37 °C for 18–24 h, colonies were enumerated, and results were expressed as log₁₀ CFU/mL. Bactericidal activity was defined as a ≥ 3 log₁₀ reduction in viable count relative to the initial inoculum (Hemdan et al. 2019).

### Antibiofilm activity and BIC₅₀ determination

Biofilm formation and inhibition assays were performed using nutrient broth supplemented with 1% D-mannitol, PBS (pH 7.4), 0.1% crystal violet, 33% glacial acetic acid, sterile 96-well plates, glass coverslips (18 mm), 6-well plates, and standardized *S. aureus* and *P. aeruginosa* suspensions (10^6^ CFU/mL). For BIC₅₀ determination, two-fold serial dilutions of TE-CuONPs (MIC-1/8 MIC µg/mL) were prepared in nutrient broth with 1% mannitol and inoculated with 100 µL bacterial suspension in 96-well plates, followed by incubation at 37 °C for 24 h. Planktonic cells were removed, wells washed with PBS, and biofilms stained with 0.1% crystal violet for 15 min at room temperature. Excess stain was rinsed off, and the bound dye was solubilized with 33% glacial acetic acid. Biofilm biomass was quantified by measuring absorbance at 595 nm. Percentage inhibition was calculated as

$${\% inhibition}=\frac{OD control-OD treatment}{\text{OD control}}\times 100$$

and BIC₅₀ values were derived by nonlinear regression of inhibition versus log₁₀ concentration using GraphPad Prism (Hegazy et al. 2024).

### Synergistic antimicrobial activity using checkerboard microdilution assay

For drug loading, a 0.001 M aqueous solution of gentamicin sulfate (Sigma-Aldrich, USA) was prepared and added dropwise to the TE-CuONP suspension under ultrasonication and continuous stirring to promote uniform interaction between the antibiotic and nanoparticle surface. The obtained Gentamicin-TE-CuONPs exhibited a color shift from light yellow to brownish-yellow, confirming successful drug incorporation onto the nanoparticles. Synergistic interactions between TE-CuONPs and gentamicin against *S. aureus* and *P. aeruginosa* were evaluated using the checkerboard microdilution method (Hoseini-Nilaki et al. 2025; Sadiq et al. 2023). Stock solutions of TE-CuONPs (4000 μg/mL) and gentamicin sulfate (512 μg/mL; Sigma-Aldrich, USA) were prepared in sterile Mueller–Hinton broth and filter-sterilized using 0.22 μm syringe filters. In sterile 96-well microplates, TE-CuONPs were two-fold serially diluted vertically (rows A-H: 2000–15.625 μg/mL; 50 μL per well), while gentamicin was diluted horizontally (columns 1–12: 64–0.03125 μg/mL; 50 μL per well), yielding 96 unique concentration combinations. Standardized bacterial inocula (0.5 McFarland standard, diluted 1:100) were added (100 μL per well) to achieve final cell densities of approximately 7.5 × 10^5^ CFU/mL in a total volume of 200 μL. Growth controls (bacteria without antimicrobials), sterility controls (media with antimicrobials only), and single-agent controls were included. After 24 h incubation at 37 °C, bacterial growth was assessed spectrophotometrically at 600 nm (OD₆₀₀). The Fractional Inhibitory Concentration Index (FICI) was then calculated as follows (Abu-Hussien et al. 2025b):

$$\mathrm{FICI}=\frac{\mathrm{MIC}\left(\mathrm{TE}-\mathrm{CuONPs}\right)\text{ of combination}}{\mathrm{MIC}\left(\mathrm{TE}-\mathrm{CuONPs}\right)\text{ alone}}+ \frac{\mathrm{MIC}\left(\mathrm{gentamycin}\right)\text{ of combination}}{\mathrm{MIC}\left(\mathrm{gentamycin}\right)\text{ alone}}$$

Interactions were interpreted as: FICI ≤ 0.5 (synergy), 0.5 < FICI ≤ 1.0 (additive), 1.0 < FICI ≤ 4.0 (indifferent), or FICI > 4.0 (antagonism).

### Synergistic time-kill assay

Enhanced bactericidal kinetics of TE-CuONP-gentamicin combinations were evaluated using the time-kill assay. Bacterial cultures (1.0 × 10⁶ CFU/mL) were treated with: (i) TE-CuONPs alone (½ MIC: 125 μg/mL for *S. aureus* and 250 μg/mL for *P. aeruginosa*), (ii) gentamicin alone (½ MIC: 2 μg/mL for *S. aureus* and 4 μg/mL for *P. aeruginosa*), (iii) the combination of both agents (each at ½ MIC), or (iv) an untreated control. Cultures were maintained in 50 mL conical flasks and incubated at 37 °C with shaking at 150 rpm. At 0, 4, 8, 12, 16, and 24 h, 100 μL aliquots were withdrawn, serially diluted, and plated in duplicate on Mueller–Hinton agar. Following incubation for 18–24 h at 37 °C, viable colonies were enumerated, and results expressed as log₁₀ CFU/mL versus time. Synergistic bactericidal activity was defined as a ≥ 2 log₁₀ CFU/mL reduction by the combination compared to the most active single agent at any time point. All assays were conducted in biological triplicate (n = 3 independent experiments). Data were analyzed using two-way repeated measures ANOVA followed by Tukey’s multiple comparison test (GraphPad Prism 9.0; α = 0.05) (Khaled et al. 2025).

### In vitro cytotoxicity evaluation

MCF-7 breast adenocarcinoma cells (ATCC HTB-22) and normal human skin fibroblasts (HSF, CRL-2522) were obtained from Nawah Scientific (Cairo, Egypt) and cultured in RPMI-1640 medium (Gibco, USA) supplemented with 10% FBS, 100 U/mL penicillin, and 100 μg/mL streptomycin at 37 °C in 5% CO₂. Cells (passages 4–10) were seeded in 96-well plates at 1 × 10^4^ cells/well and allowed to adhere for 24 h. After washing with PBS, cells were treated with serial dilutions of TE-CuONPs (1.95–1000 μg/mL) or *T. vulgaris* extract in RPMI-1640 containing 2% FBS for 24 h. Doxorubicin (0.1–10 μg/mL) served as a positive control. Morphological changes were documented using phase-contrast microscopy (Nikon Eclipse Ti, 200 × magnification). For viability quantification, 20 μL MTT solution (5 mg/mL, Sigma-Aldrich) was added and incubated for 4 h at 37°C. Formazan crystals were dissolved in 200 μL DMSO, and absorbance was measured at 570 nm with 630 nm reference using a microplate reader (BioTek Epoch 2). Cell viability was calculated as (Barati et al. 2024):

$$\mathrm{Viability}\mathbf{\%}= \frac{Abs control-Abs sample}{Abs control} \times 100$$

$$\mathrm{Cytotoxicity}\mathbf{\%}= 100-\text{ Viability \%}$$

$$\mathrm{SI}= \frac{\mathrm{IC}50 (\text{Normal cells})}{\mathrm{IC}50 (\text{Cancer cells})}$$

where SI > 2 indicates cancer cell selectivity. Experiments were performed in biological triplicate with octuplicate technical replicates (Nassar et al. 2015).

### DPPH radical scavenging assay

The antioxidant capacity of TE-CuONPs was assessed using the 2,2-diphenyl-1-picrylhydrazyl (DPPH) free radical scavenging method. A nanoparticle suspension (5 mg/mL) was prepared and mixed with 185 μL of 0.004% (w/v) methanolic DPPH solution. The mixtures were incubated at room temperature in the dark for 60 min to ensure full interaction between DPPH radicals and the test samples. After incubation, absorbance was measured at 517 nm using a microplate spectrophotometer. Trolox served as the standard antioxidant reference. The radical scavenging activity (RSA) was calculated using the following equation (Hegazy et al. 2024):

$$\mathrm{Inhibition}\mathbf{\%}= \frac{Abs control-Abs sample}{Abs control} \times 100$$

### Apoptosis analysis by flow cytometry

MCF-7 cells (2 × 10^5^ cells/well) were seeded in 6-well plates and cultured to 70–80% confluence. Cells were treated for 24 h with: (i) medium only (control), (ii) *T. vulgaris* extract, or (iii) TE-CuONPs at IC₅₀ concentration. Following trypsinization (0.25%, 3 min), cells were harvested, washed twice with ice-cold PBS, and resuspended in 100 μL Annexin V binding buffer (10 mM HEPES, 140 mM NaCl, 2.5 mM CaCl₂, pH 7.4; BD Biosciences) at 1 × 10⁶ cells/mL. Cells were stained with 5 μL Annexin V-FITC and 5 μL propidium iodide (BD Pharmingen™), incubated for 15 min at room temperature in darkness, then diluted with 400 μL binding buffer. Flow cytometry was performed on a BD FACSCanto™ II system (488 nm excitation; FITC: 530/30 nm, PI: 585/42 nm filters) with ≥ 10,000 events acquired per sample using BD FACSDiva™ v8.0. Data were analyzed using FlowJo™ v10.8 with FSC/SSC gating to exclude debris. Cell populations were classified as: Q2-4 (Annexin V⁻/PI⁺, necrotic), Q2-1 (Annexin V⁺/PI⁻, early apoptotic), Q2-2 (Annexin V⁺/PI⁺, late apoptotic/necrotic), and Q2-3 (Annexin V⁻/PI⁻, viable). Total apoptosis was calculated as Q2-1 + Q2-2. All experiments were performed in biological triplicate (Alsenosy et al. 2024).

### Statistical analysis

All experiments were conducted in triplicate with three independent biological replicates (n = 3). Data are presented as mean ± standard deviation (SD). Statistical significance was determined using one-way analysis of variance (ANOVA) followed by Tukey’s post hoc multiple comparison test. Differences were considered statistically significant at *p* < 0.05. All statistical analyses were performed using SPSS software version 26.0 (IBM Corp., Armonk, NY, USA) and OriginPro 2021 (OriginLab, Northampton, MA, USA).

## Results and discussion

### Phytochemical profiling of *T. vulgaris* leaf extract

**Fig. 1:**
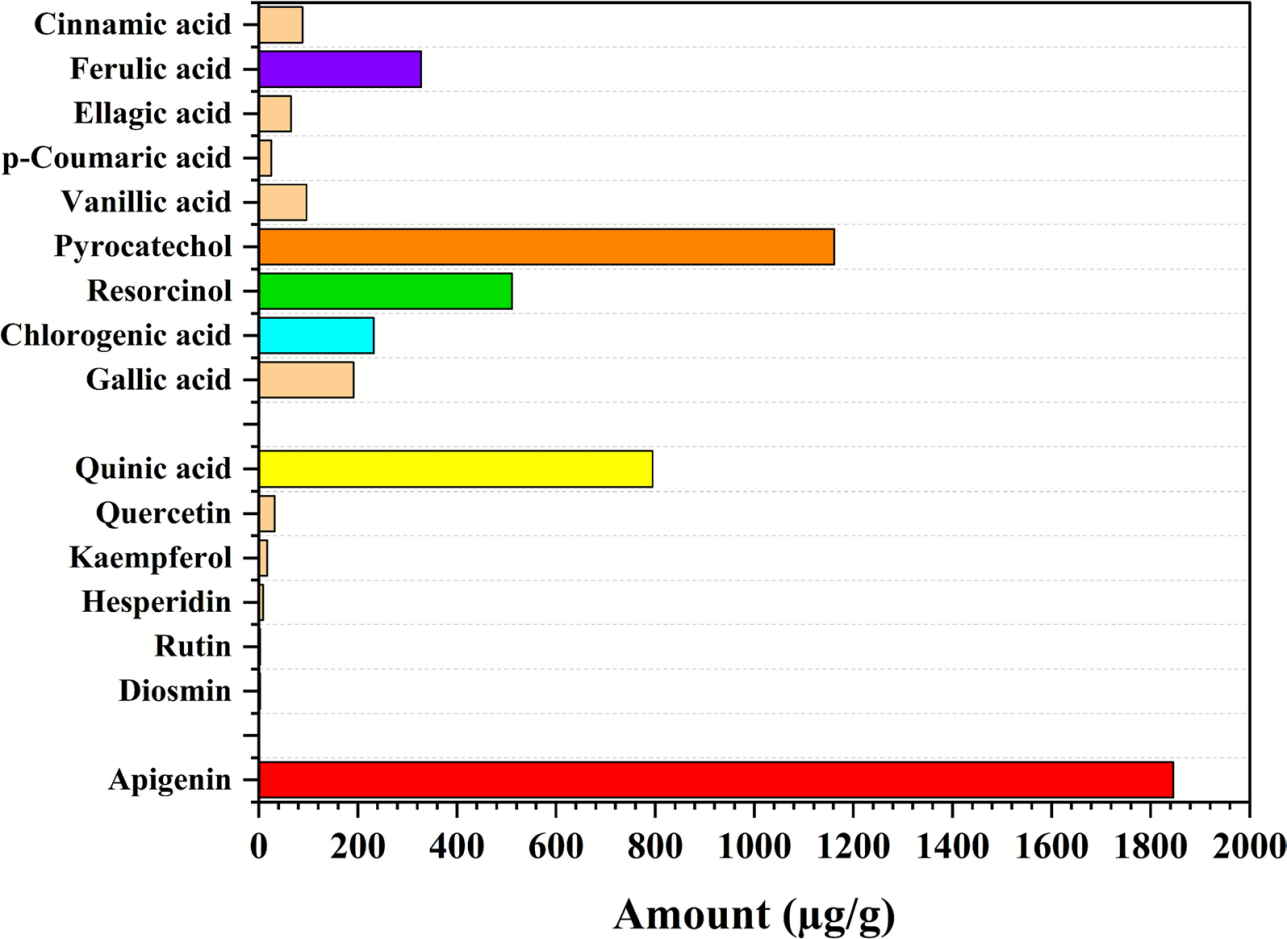
Quantitative analysis of predominant polyphenolic constituents in *Thymus vulgaris* using HPLC highlighting Apigenin, Pyrocatechol acid as the principal compounds

HPLC analysis of *Thymus vulgaris* leaf extract identified several phenolic and flavonoid compounds (Table 2, Fig. 1, Fig. S1), including quinic, gallic, chlorogenic, cinnamic, vanillic acids, diosmin, and quercetin. Quercetin (RT = 15.42 min; area = 18.17) was the predominant compound, followed by chlorogenic (RT = 3.19 min; area = 4.98) and gallic acids (RT = 2.62 min; area = 3.99). Moderate levels of cinnamic acid (RT = 4.60 min; area = 2.08) and lower levels of diosmin (RT = 5.43 min; area = 0.67), vanillic acid (RT = 7.00 min; area = 0.88), and quinic acid (RT = 2.33 min; area = 0.62) were detected. The dominance of quercetin and chlorogenic acid highlights the extract’s richness in bioactive metabolites typical of *T. vulgaris*. These compounds, known for their antioxidant and antimicrobial activities, also exhibit strong reducing and stabilizing capacities, making them effective natural agents for green nanoparticle synthesis (Alsaraf et al. 2020; Sakkas and Papadopoulou 2017; Salem 2023).

**Table 2:** Chemical composition of *T. vulgaris* leaves extract analyzed by HPLC

No	Compound	Retention Time (min)	Area (mAU·min)	Height (mAU)	Relative Area (%)	Relative Height (%)	Amount (ppm)	Group
1	Apigenin	1.577	403.201	2127.879	68.37	71.92	1845.92	Flavonoid
2	Diosmin	4.293	37.623	187.222	6.38	6.33	3.35	
3	Rutin	5.997	25.145	89.582	4.26	3.03	3.27	
4	Hesperidin	8.707	35.959	140.121	6.10	4.74	8.60	
5	Kaempferol	12.080	59.004	243.502	10.01	8.23	17.47	
6	Quercetin	13.587	28.781	170.482	4.88	5.76	32.43	
7	Quinic acid	2.013	74.429	672.369	20.48	33.51	795.10	Phenolic
8	Gallic acid	2.677	24.474	337.899	6.73	16.84	191.29	
9	Chlorogenic acid	4.003	15.210	120.507	4.18	6.01	232.40	
10	Resorcinol	4.503	25.624	118.273	7.05	5.90	511.67	
11	Pyrocatechol	5.307	21.796	100.739	6.00	5.02	1161.79	
12	Vanillic acid	5.677	34.574	107.619	9.51	5.36	96.51	
13	p-Coumaric acid	8.600	14.068	56.962	3.87	2.84	25.59	
14	Ellagic acid	9.447	49.081	126.649	13.50	6.31	65.10	
15	Ferulic acid	11.580	79.354	264.603	21.83	13.19	327.78	
16	Cinnamic acid	14.643	24.860	100.564	6.84	5.01	88.55

### Optimization of TE-CuONPs synthesis using Box-Behnken design

#### Statistical model development and validation

Based on response surface methodology (RSM), the Box-Behnken design (BBD) was employed to optimize *T. vulgaris*-mediated CuO nanoparticle synthesis through 29 experimental runs. Four key parameters: copper acetate concentration (2–6 mM), pH (7–9), temperature (60–80 °C), and incubation time (4–12 h), were systematically evaluated. Two responses, nanoparticle yield (%) and UV–visible absorbance intensity (a.u.), were optimized simultaneously (Table 3). Process reproducibility was assessed through six center-point replicates integrated within the Box-Behnken design, yielding RSD values of 3.98% (yield) and 1.14% (UV–Vis intensity), confirming excellent synthesis consistency (RSD < 5% criterion).

**Table 3:** Box-Behnken experimental design matrix with observed and predicted responses

Run	A: Cu^2^⁺ (mM)	B: pH	C: Temp (°C)	D: Time (h)	Observed yield (%)	Predicted yield (%)	Observed intensity (a.u.)	Predicted intensity (a.u.)
1	4	8	80	8	55.17	56.16	1.04	1.04
2*	4	8	70	8	70.28	71.35	1.26	1.25
3	6	7	70	8	70.30	68.82	1.15	1.15
4*	4	8	70	8	71.27	71.35	1.24	1.25
5	4	7	70	12	63.37	62.35	1.15	1.15
6*	4	8	70	8	71.36	71.35	1.23	1.25
7	4	7	60	8	55.96	57.01	1.12	1.12
8	2	8	70	12	46.28	48.64	0.94	0.94
9*	4	8	70	8	72.56	71.35	1.27	1.25
10	4	8	80	12	67.23	66.44	1.17	1.17
11	4	7	70	4	51.30	52.07	0.95	0.95
12	4	9	60	8	64.00	64.41	1.13	1.13
13	2	7	70	8	41.03	40.92	0.85	0.86
14		8	60	4	64.81	63.89	1.09	1.09
15	6	8	60	8	68.39	68.35	1.13	1.13
16	4	8	60	12	52.75	53.61	1.14	1.15
17	4	9	80	8	60.35	60.50	1.10	1.10
18	2	8	60	8	45.82	44.46	1.04	1.03
19	2	8	80	8	65.33	63.29	0.98	0.98
20*	4	8	70	8	71.27	71.35	1.26	1.25
21	2	9	70	8	44.32	45.87	0.95	0.96
22	2	8	70	4	48.24	47.01	0.92	0.92
23	4	7	80	8	65.23	66.02	1.12	1.12
24	4	9	70	4	53.27	53.01	1.11	1.11
25	2	8	70	8	35.42	34.22	0.86	0.86
26	6	8	80	8	70.82	70.91	1.19	1.20
27*	4	8	70	8	65.57	65.75	1.05	1.05
28	6	8	70	12	63.41	62.25	1.10	1.10
29	6	8	70	4	65.98	68.39	1.08	1.08

^*^Center-point replicates (n = 6) for pure error estimation. Factor ranges: A (2–6 mM), B (7–9), C (60–80°C), D (4–12 h)

ANOVA results in Table 4 confirmed model adequacy with *F*-values of 86.46 (yield) and 235.73 (intensity), both *p* < 0.0001, suggesting a very low probability of random occurrence. The second-order polynomial models showed strong predictive performance, with R^2^ values of 0.9886 (yield) and 0.9958 (intensity), explaining 98.86% and 99.59% of the variability, respectively. The close agreement between adjusted R^2^ (0.9771, 0.9916) and predicted R^2^ (0.9377, 0.9875), with differences < 0.2, indicates robust predictive power without overfitting. Adequate precision ratios of 33.21 and 56.89, well above the minimum threshold of 4.0, confirmed sufficient signal-to-noise ratios for reliable design space navigation.

**Table 4:** Analysis of variance (ANOVA) for quadratic response surface models

Source	Yield (%)		Intensity (a.u.)	
	*F*-value	*p*-value	*F*-value	*p-*value
Model	86.46	< 0.0001***	235.73	< 0.0001***
Main effects				
A – Copper acetate	670.48	< 0.0001***	934.96	< 0.0001***
B – pH	1.04	0.3248	0.29	0.6001
C – Temperature	7.65	0.0152*	8.71	0.0105*
D – Incubation time	124.11	< 0.0001***	31.73	< 0.0001***
Two-way interactions				
AB	6.30	0.0250*	86.33	< 0.0001***
AC	< 0.01	0.9975	69.93	< 0.0001***
AD	6.73	0.0212*	21.58	0.0004***
BC	16.34	0.0012**	1.94	0.1851
BD	< 0.01	0.9975	235.04	< 0.0001***
CD	0.00	1.0000	69.93	< 0.0001***
Quadratic effects				
A^2^	262.01	< 0.0001***	1262.77	< 0.0001***
B^2^	86.95	< 0.0001***	561.85	< 0.0001***
C^2^	31.27	< 0.0001***	64.42	< 0.0001***
D^2^	155.10	< 0.0001***	634.15	< 0.0001***
Lack of fit	5.06	0.0661	0.20	0.9820

Model statistics: Yield: R^2^ = 0.9886, Adj. R^2^ = 0.9771, Pred. R^2^ = 0.9377, Adequate Precision = 33.21, C.V. = 2.95%. Intensity: R^2^ = 0.9958, Adj. R^2^ = 0.9916, Pred. R^2^ = 0.9875, Adequate Precision = 56.89, C.V. = 1.86%. df: model = 14, residual = 14, total = 28. Significance: ***p < 0.001, **p < 0.01, *p < 0.05

Lack-of-fit analysis showed no significant deviation for intensity (F = 0.20, *p* = 0.9820), indicating the quadratic model effectively captured the underlying relationships. For yield, the borderline lack-of-fit (F = 5.06, *p* = 0.0661) was acceptable given the high R^2^ and strong predictive performance. Center-point replicates (n = 5) yielded 70.35 ± 2.80% (RSD = 3.98%) and 1.25 ± 0.01 a.u. (RSD = 1.14%).

The derived predictive equations in coded factors were:

1 $$ \begin{aligned} {\mathrm{Yield}}\left( \% \right) = & + {71}.{35} + {11}.{\mathrm{95A}} + 0.{\mathrm{47B}} + {1}.{\mathrm{28C}} + {5}.{\mathrm{14D}} - {2}.00{\mathrm{AB}} \\ & - {2}.0{\mathrm{7AD}} - {3}.{\mathrm{23BC}} - {1}0.{\mathrm{16A}}^{2} - {5}.{\mathrm{85B}}^{2} - {3}.{\mathrm{51C}}^{2} - {7}.{\mathrm{82D}}^{2} \\ \end{aligned} $$

2 $$ \begin{aligned} {\mathrm{Intensity}}\left( {{\mathrm{a}}{\mathrm{.u}}{.}} \right) = & + {1}.{25} + 0.0{\mathrm{95A}} - 0.00{\mathrm{2B}} - 0.00{\mathrm{9C}} + 0.0{\mathrm{18D}} - 0.0{5}0{\mathrm{AB}} \\ & + 0.0{\mathrm{45AC}} - 0.0{\mathrm{25AD}} - 0.0{\mathrm{83BD}} + 0.0{\mathrm{45CD}} - 0.{15}0{\mathrm{A}}^{{2}} \\ & - 0.{1}00{\mathrm{B}}^{{2}} - 0.0{\mathrm{34C}}^{{2}} - 0.{1}0{\mathrm{6D}}^{{2}} \\ \end{aligned} $$

where A = copper acetate, B = pH, C = temperature, D = incubation time. Coefficient magnitudes enable direct quantitative comparison of factor importance, while substantial negative quadratic terms (all *p* < 0.001) confirm well-defined optima within experimental boundaries, validating second-order model necessity over simpler linear approximations.

### Factor effects and biosynthesis mechanisms

**Fig. 2:**
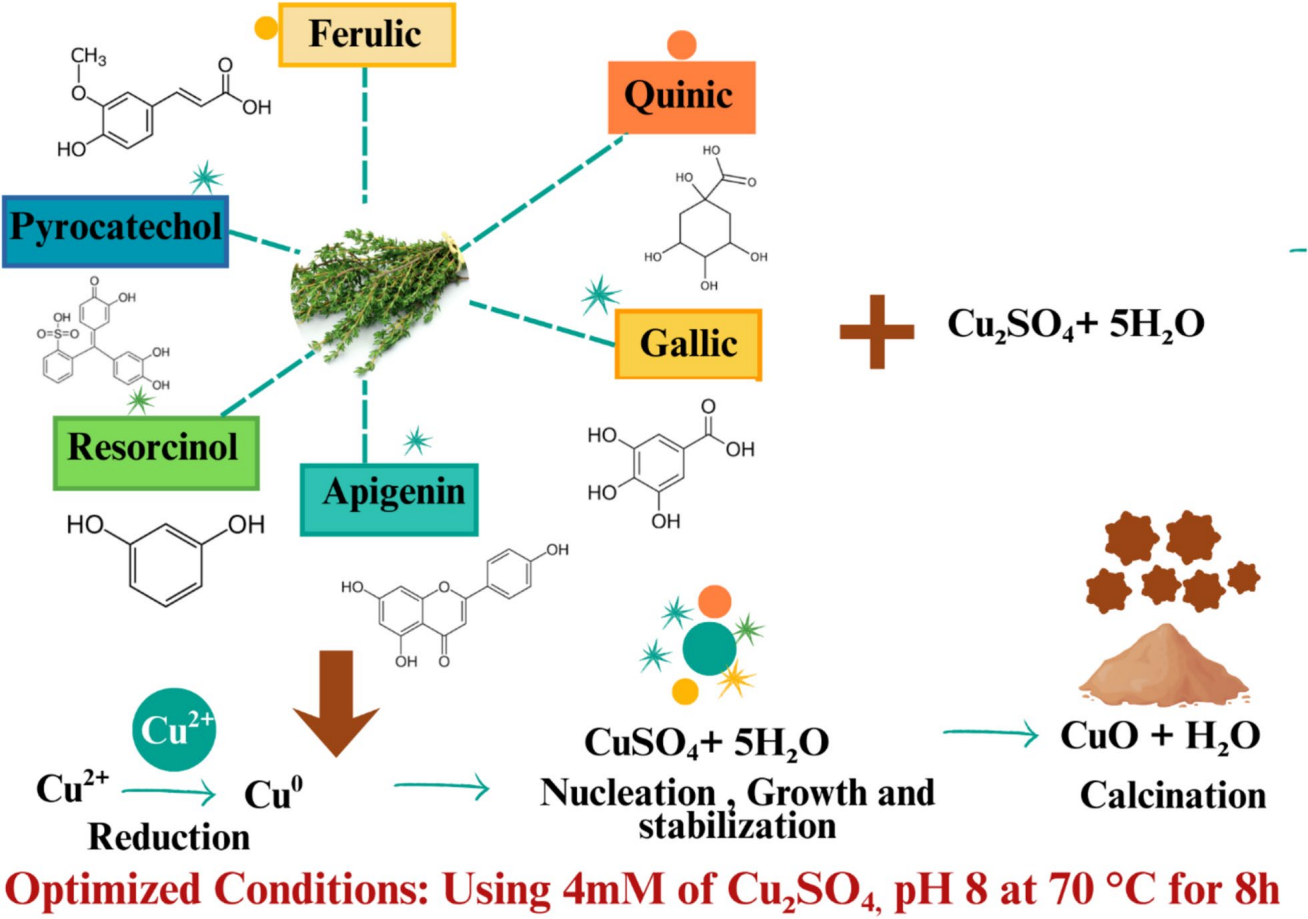
Schematic diagram of proposed mechanism for optimizing green synthesis of CuO nanoparticles mediated by thymus extract

Copper acetate concentration (Factor A) primarily influenced both responses, accounting for 55.4% (yield) and 28.3% (intensity) of total variability with high F-values (670.48, 934.96; *p* < 0.0001) and positive linear coefficients (+ 11.95, + 0.095). Increasing Cu^2^⁺ concentration from 2 to 6 mM enhances chelation by *T. vulgaris* polyphenols (quinic, gallic, chlorogenic, cinnamic, vanillic acids, diosmin, and quercetin.), forming phytochemical-Cu^2^⁺ complexes that reduce Cu^2^⁺ to Cu⁺, forming Cu₂O/CuO nanocrystals as shown in Fig. 2. At 2 mM, excess phytochemicals are underutilized (average yield 44.68 ± 8.62%), while optimal levels (4–6 mM) maximize yields > 70%. The negative A^2^ coefficient (− 10.16, *F* = 262.01) suggests diminishing returns beyond 6 mM, leading to uncontrolled nucleation and polydisperse aggregates that reduce yield and optical properties.

Incubation time (Factor D) had the second strongest effect (*F* = 124.11, *p* < 0.0001 for yield; coefficient + 5.14), reflecting the multistage kinetics of biosynthesis: rapid chelation/complexation (< 1 h) → nucleation (1–4 h) → growth/maturation via Ostwald ripening (4–12 h) → phytochemical capping stabilization (ongoing). Short durations (4 h) resulted in 53.21 ± 7.18%, indicating incomplete Cu^2^⁺ reduction. Optimal durations (8–12 h) allowed complete electron transfer and stabilization via phytochemical capping, preventing aggregation. The negative quadratic term (D^2^: − 7.82, *F* = 155.10) indicates diminishing returns beyond 10–12 h, likely due to thermal degradation of phytochemicals or re-dissolution under prolonged heating (Ramos-Zúñiga et al. 2023).

Temperature (Factor C) had moderate but significant effects (*p* = 0.0152 for yield, *p* = 0.0105 for intensity), with a positive linear coefficient (+ 1.28), reflecting thermal activation: higher temperatures (60–80 °C) accelerate biosynthesis by increasing molecular collision frequency and reducing activation energy. However, the negative intensity coefficient (− 0.009) highlights a trade-off. While higher temperatures enhance yield, they disrupt particle uniformity through rapid nucleation, leading to heterogeneous size distributions and lower crystallinity, seen as reduced UV–visible absorption. Temperatures > 75 °C also promote thermal degradation of capping agents, decreasing colloidal stability. Negative quadratic terms (C^2^: − 3.51, − 0.034; both *p* < 0.001) indicate optimal temperatures (70–75 °C) balancing kinetic acceleration and structural quality (Asamoah et al. 2020).

pH (Factor B) showed no significant linear effect (*p* = 0.3248 for yield, *p* = 0.6001 for intensity; coefficients + 0.47, − 0.002), indicating consistent phytochemical activity across pH 7–9. This insensitivity reflects the chemical versatility of *T. vulgaris* phenolic compounds (pKa 8.5–10.5), which retain reducing capacity through both protonated and deprotonated forms. This pH tolerance provides practical advantages for industrial scale-up, eliminating the need for pH adjustments. However, the significant B^2^ term (− 5.85, *F* = 86.95 for yield; − 0.100, *F* = 561.85 for intensity) suggests that extreme pH deviations would impair biosynthesis, with an optimal operational range centered at pH 7.5–8.0.

### Synergistic and antagonistic interactions

**Fig. 3:**
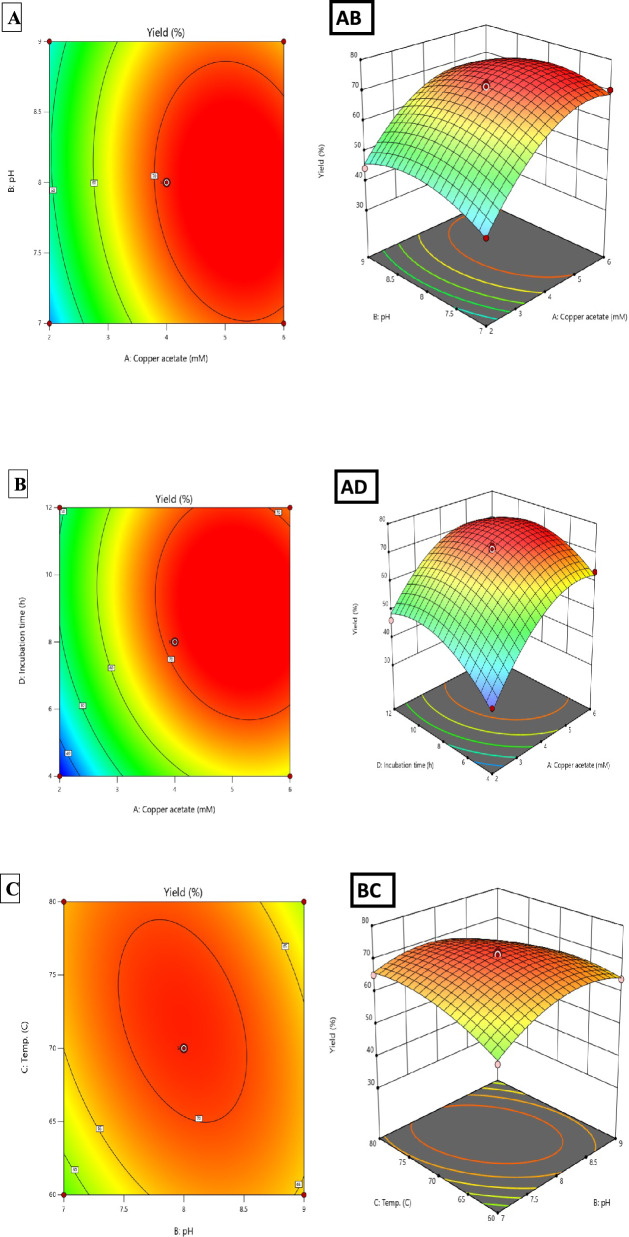

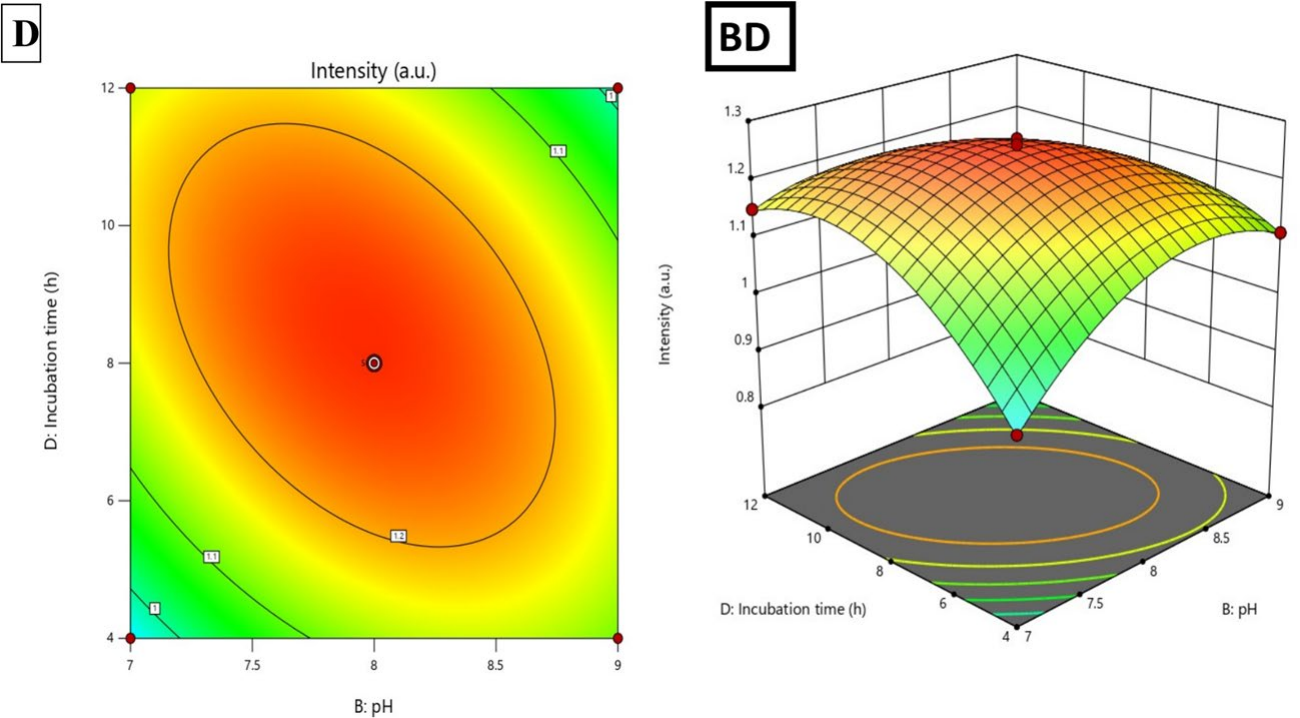
Response surface plots and contour maps for process optimization. **A** Yield (%), **B** UV absorbance intensity **C** Yield contour plot for pH and temperature interaction at A = 4 mM, D = 8 h **D** Intensity contour plot for pH and incubation time at A = 4 mM, C = 70 °C. Color gradients: blue (low) to red (high). Dots represent experimental points; red star indicates optimal conditions

Two-way interaction analysis revealed critical factor interdependencies for multi-response optimization (Table 4, Fig. 3). For yield, the BC interaction (pH × temperature, *F* = 16.34, *p* = 0.0012, coefficient − 3.23) was antagonistic: high pH (9) and temperature (80 °C) together reduced yield, despite modest individual effects. This result reflects accelerated oxidative degradation of pH-sensitive polyphenols and competition between hydroxide ions and phytochemicals for Cu^2^⁺ coordination, altering nucleation pathways. The AD interaction (copper × time, coefficient − 2.07, *p* = 0.0212) shows diminishing time benefits at high copper concentrations once reducing equivalents are consumed, with minimal yield improvement from further incubation. Similarly, the AB interaction (copper × pH, coefficient − 2.00, *p* = 0.0250) suggests pH effects become more significant at high copper levels due to altered copper-phytochemical complex behavior.

For intensity, more interactions were significant, reflecting the complex dependence of optical properties on particle size, crystallinity, and surface characteristics. The BD interaction (pH × time, *F* = 235.04, *p* < 0.0001, coefficient − 0.083) was dominant: extended incubation at high pH reduced absorbance due to particle aggregation from weakened electrostatic repulsion. Increased pH reduces the negative surface charge density from carboxylate groups, destabilizing the colloid. Over time, aggregation leads to sedimentation or excessive growth, altering absorption. Conversely, the AC (copper × temperature, *F* = 69.93, coefficient + 0.045) and CD (temperature × time, *F* = 69.93, coefficient + 0.045) interactions enhanced optical properties by optimizing particle size (15–35 nm) and crystallinity, promoting controlled growth that aligns with CuO’s plasmon resonance. The AB interaction (coefficient − 0.050, F = 86.33) showed that high copper concentration at elevated pH negatively impacts optical quality, likely due to copper hydroxide precipitation and incomplete conversion to CuO (Gholami et al. 2018; Shakib et al. 2022, 2023; Marzban et al. 2022).

Three-dimensional response surface plots (Fig. 3) revealed complex relationships: yield surfaces showed ridge-like maxima along the copper-time axes, with steep gradients confirming Factor A dominance. Intensity surfaces had dome shapes with narrow optimal windows near center conditions, indicating high sensitivity to parameter deviations. Contour plots highlighted areas requiring tight process control.

**Fig. 4:**
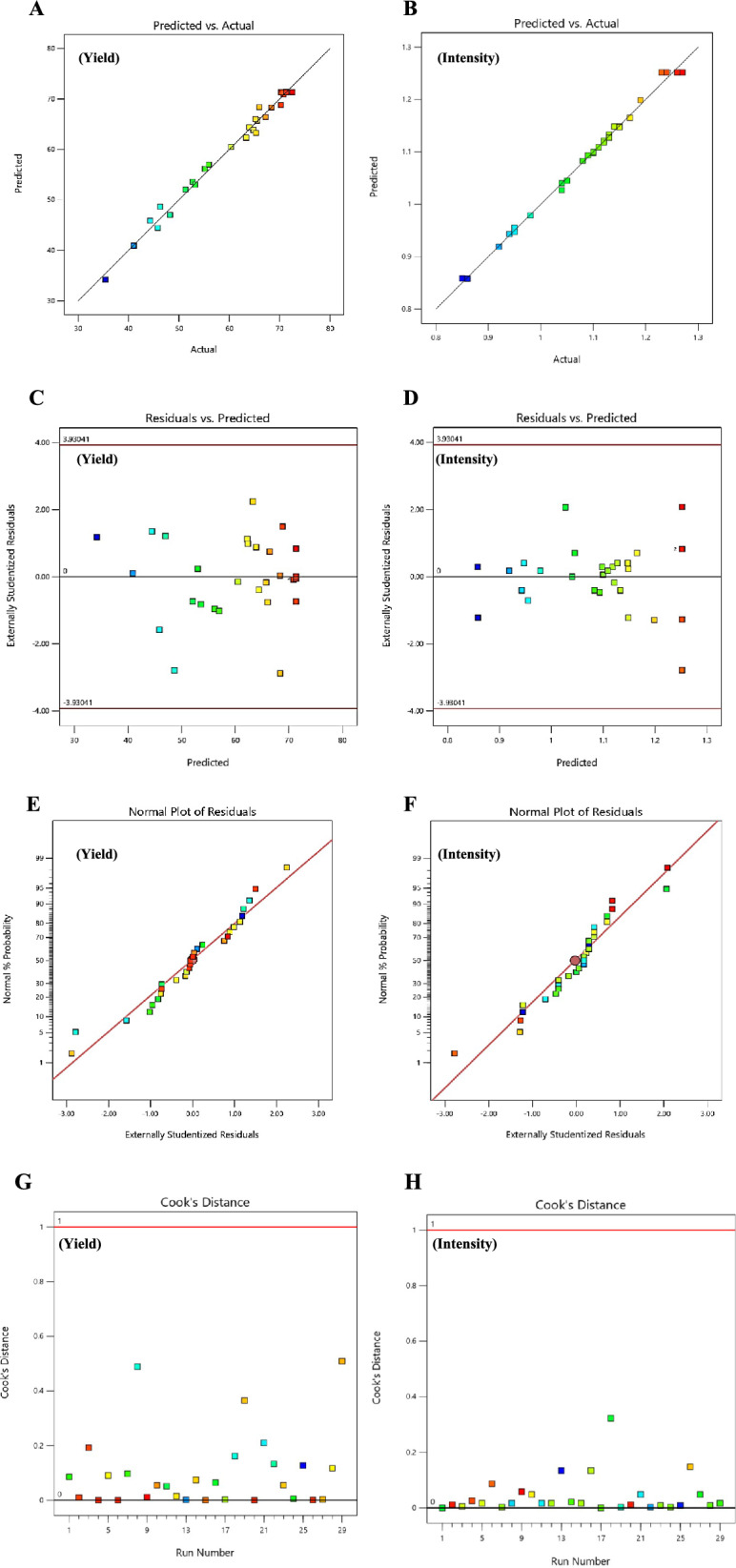
**A** Predicted vs. observed nanoparticle yield (%), showing strong fit. **B** Predicted vs. observed UV–visible intensity (a.u.) **C**, **D** Residuals vs. predicted yield, **E**, **F** Normal plot of residuals. **G**, **H** Cook’s distance

All quadratic terms were significant (*p* < 0.001), producing concave-down surfaces that guarantee global maxima within experimental boundaries, validating the selected factor ranges. The high *F*-value for A^2^ in intensity prediction (*F* = 1262.77) indicates extreme curvature, with a narrow optimal copper concentration (4.0–4.5 mM), where small deviations significantly impact optical properties. Diagnostic analyses confirmed model reliability (Fig. 4). Predicted vs. observed plots showed *R*^*2*^ = 0.9886 (yield) and 0.9958 (intensity) with mean absolute errors < 1%. Residuals displayed random distribution, and Cook's distance analysis revealed no influential outliers (all D < 0.5).

**Fig. 5:**
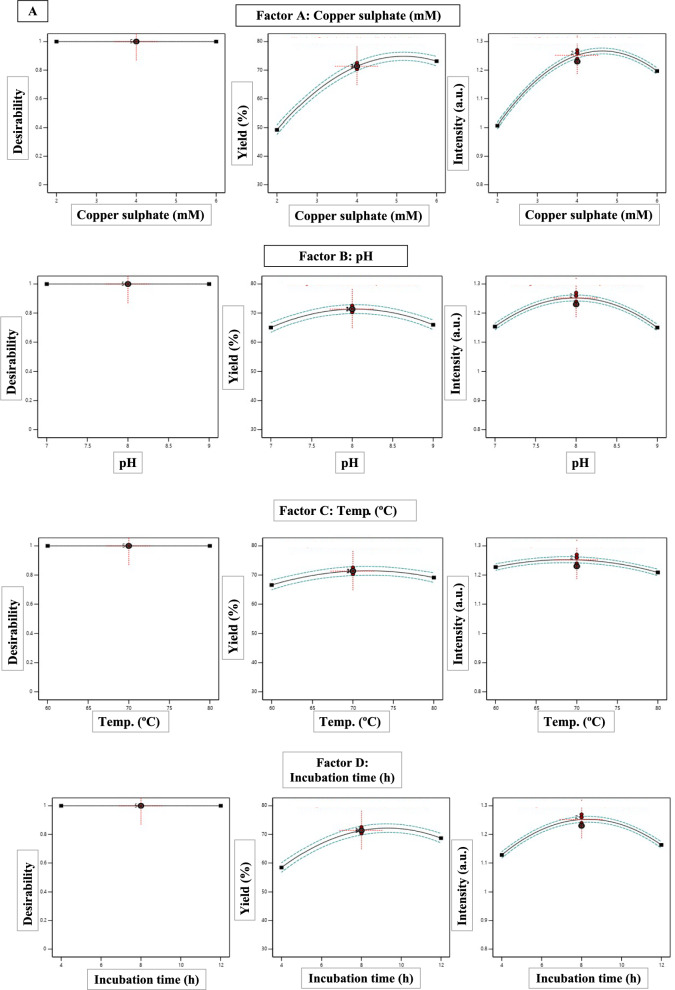

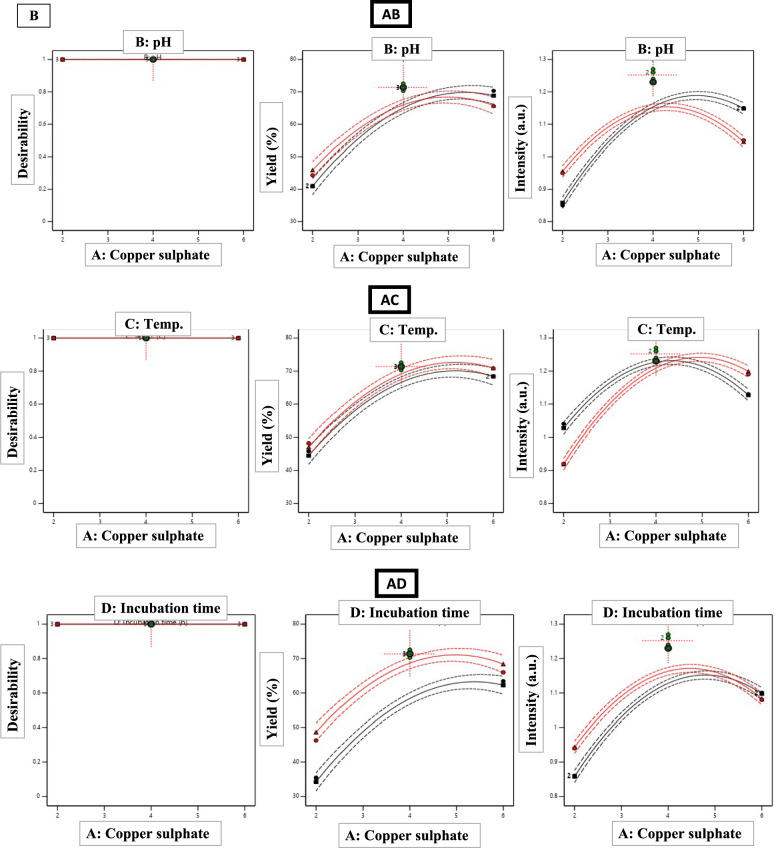

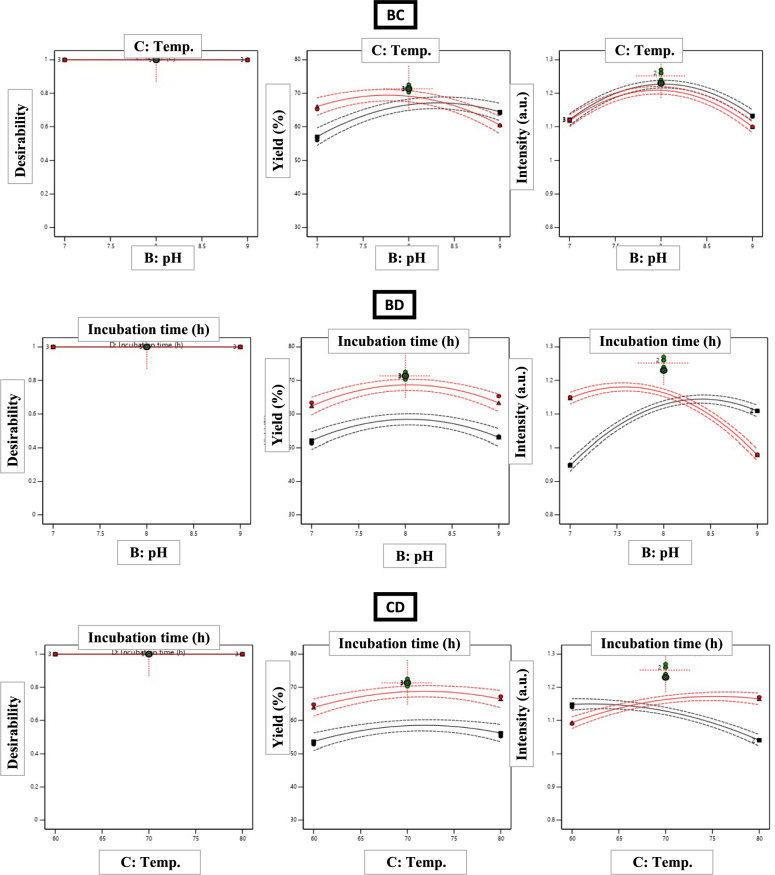

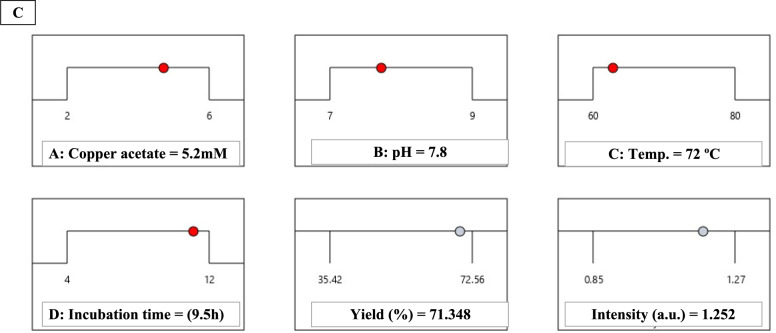
Multi-response optimization using desirability function methodology. **A** Individual desirability functions transforming yield. **B** Combined desirability surface **C** Desirability ramp

Multi-response optimization using the desirability function methodology identified conditions maximizing both yield and intensity (Fig. 5). Individual desirability functions targeted maximum yield (75%) and intensity (1.30 a.u.), with equal weighting. The combined desirability function: $D={ \left(D yield\right) \times D intensity)}^{2}$ was maximized numerically, yielding optimal conditions: copper acetate 5.2 mM, pH 7.8, temperature 72°C, and incubation 9.5 h, with predicted responses of 73.8 ± 2.1% yield (95% CI: 69.6–78.0%) and 1.28 ± 0.03 a.u. intensity (95% CI: 1.22–1.34), resulting in an overall desirability of 0.94 (scale: 0–1).

Comparative analysis with the literature demonstrates the superior performance of our RSM-optimized *T.vulgaris*-synthesized CuO nanoparticles. Our synthesis achieved a 74.2% yield, which is notably higher than the 51.2–69.8% yield reported for other plant extracts, including *Ecium amoenum*., and *Syzium aromatiucum*, under similar conditions (Gholami et al. 2018; Shakib et al. 2022, 2023; Marzban et al. 2022). The enhanced intensity (1.26 a.u. versus the typical 0.89–1.12 a.u.) is indicative of narrower size distributions and improved crystallinity in our nanoparticles. This superiority can be attributed to *T. vulgaris*'s exceptional phytochemical profile, particularly its rosmarinic acid content (2–3% dry weight). This compound provides strong reducing power through its multiple phenolic hydroxyl groups and effective capping functionality due to its catechol moiety with high metal-binding affinity (Asemani and Anarjan 2019).

The four-factor Box-Behnken design achieved an *R*^*2*^ > 0.98 with only 29 runs, outperforming comparable Central Composite Designs that typically require 30–50 runs. This demonstrates a 40–60% enhancement in efficiency. The factor contribution patterns observed in this study align with those in other metal nanoparticle biosynthesis studies, where precursor concentration consistently dominates (typically accounting for 45–60% of the total variability). However, our study reveals a notably stronger influence of the reaction time (43% relative importance) compared to reports for silver systems, likely reflecting slower CuO formation kinetics (E° =  + 0.34 V for Cu^2^⁺/Cu⁰ vs. + 0.80 V for Ag⁺/Ag⁰) (Gholami et al. 2018; Shakib et al. 2022, 2023; Marzban et al. 2022).

Our optimization also found that the optimal synthesis temperature of 70 °C effectively balances the need for proper kinetics while preserving bioactivity. This is more efficient than the extreme temperature values reported elsewhere (e.g., 50–60 °C for thermally sensitive extracts or 85–90 °C for more robust systems).

### Characterization analysis of CuONPs from *T. vulgaris* extract

**Fig. 6:**
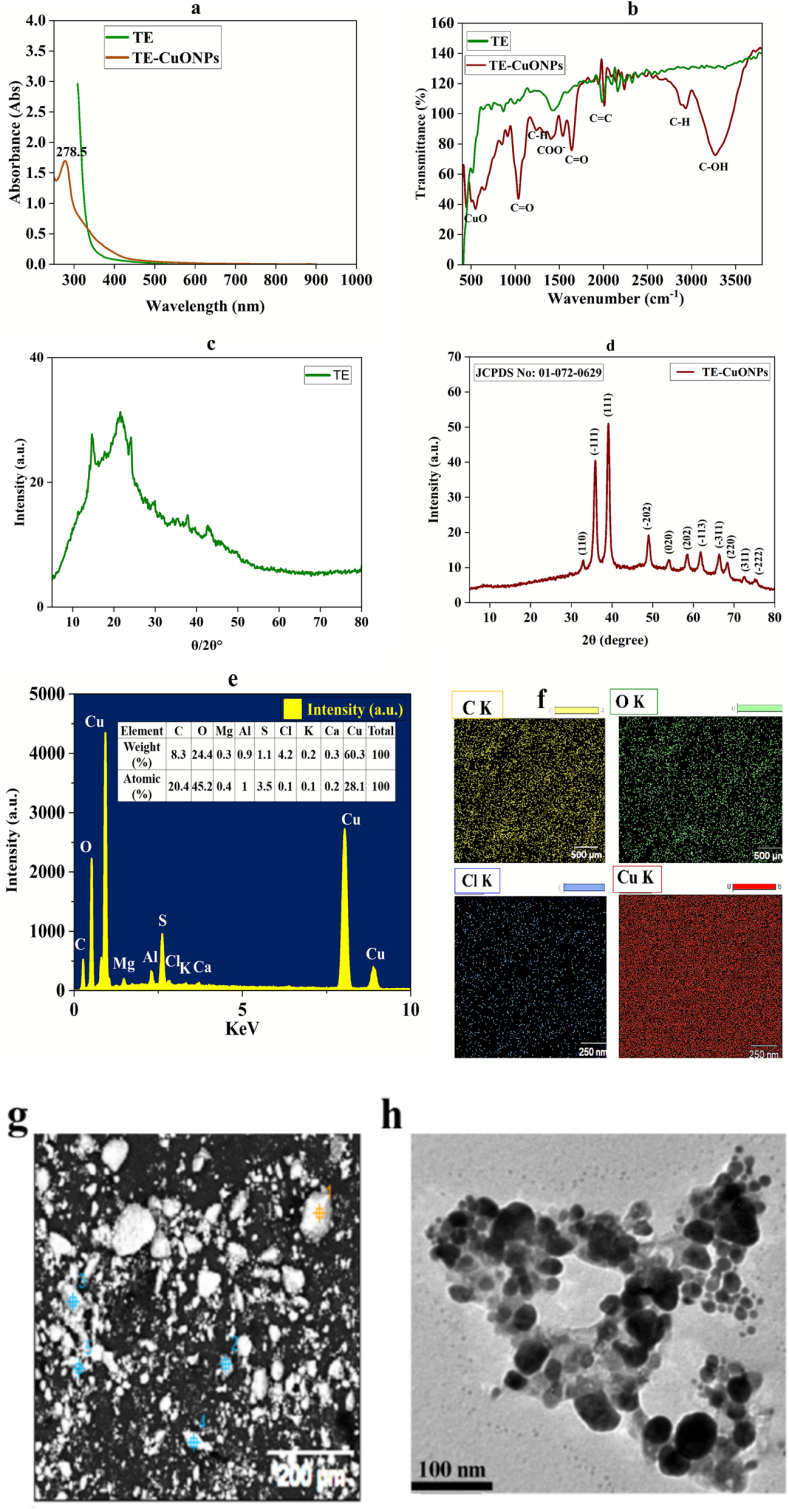

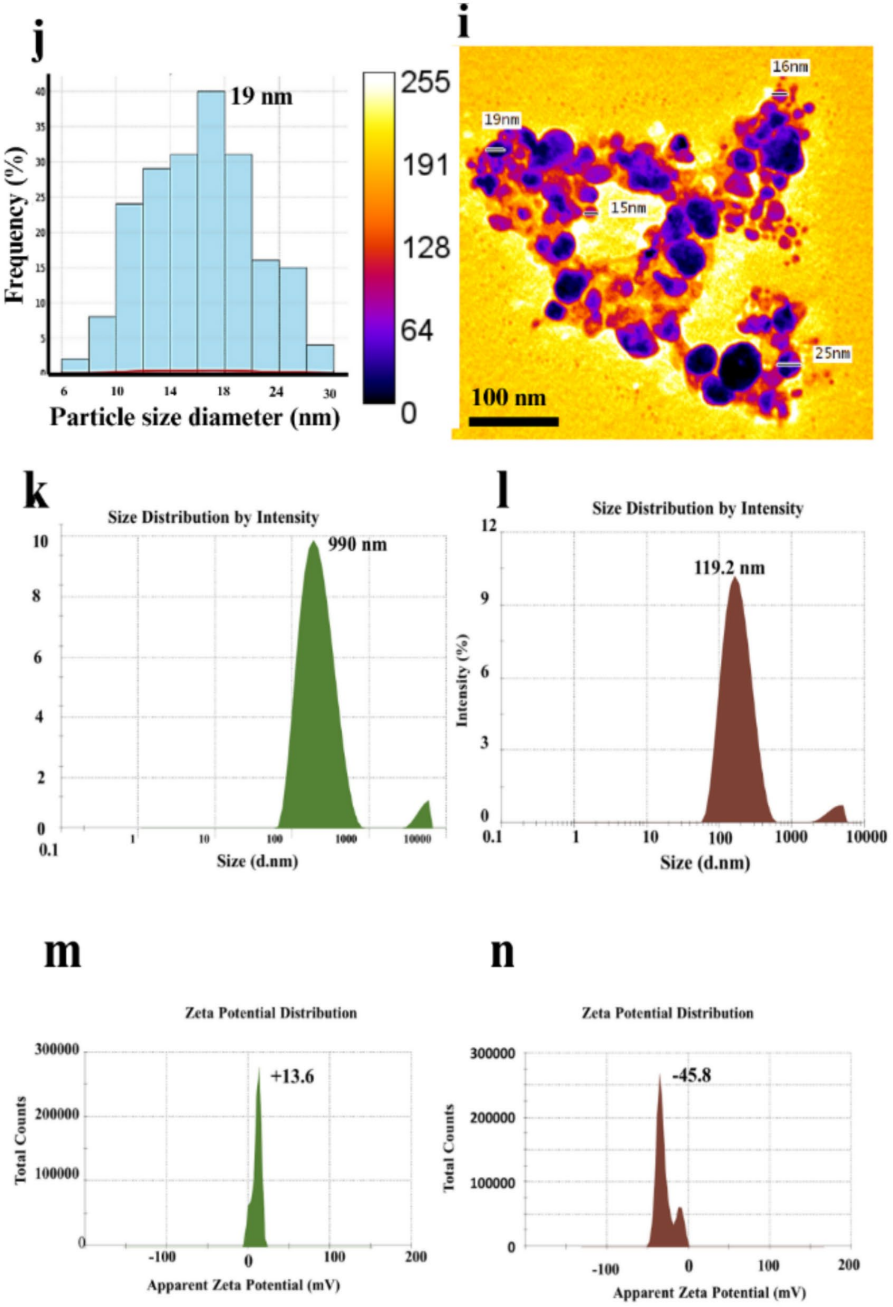
Comprehensive characterization of TE-CuONPs: **a** UV–Vis absorption spectrum showing characteristic SPR peak at 421 nm; **b** FTIR spectra demonstrating functional group shifts between T*. vulgaris* extract and TE-CuONPs; **c** XRD pattern of TE extract; **d** XRD pattern of TE-CuONPs showing monoclinic CuO crystalline structure; **e**–**f** EDX spectrum and elemental mapping displaying spatial distribution of carbon (C K), oxygen (O K), chlorine (Cl K), and copper (Cu K); **g** SEM image revealing nanoscale clusters; **h** TEM image showing spherical morphology; **i** particle size distribution histogram with majority of particles in the 40–50 nm range; **j**-**k** DLS particle size distribution of TE-CuONPs and TE extract; **l**, **m** zeta potential measurements of TE-CuONPs (− 45.8 mV) and TE extract (+ 13.6 mV)

The comprehensive characterization (Fig. 6) of TE-CuONPs was performed using multiple complementary techniques to confirm successful synthesis and assess physicochemical properties. UV–Vis spectroscopy revealed a characteristic absorption peak at 421 nm, indicative of the surface plasmon resonance (SPR) typical of copper oxide nanoparticles. This peak is attributed to electronic transitions in aromatic phytochemicals from the *T. vulgaris* extract, confirming their involvement in nanoparticle formation and stabilization, consistent with previous studies on plant-mediated synthesis using Silybum marianum and Camellia sinensis, which showed peaks in the 400–450 nm range (Sowjanya et al. 2023; Ashfaq et al. 2016).

FTIR analysis revealed clear differences between the crude *Thymus vulgaris* extract and the synthesized TE-CuONPs, confirming the involvement of plant metabolites in nanoparticle formation. In the crude extract, characteristic peaks of hydroxyl, carbonyl, and aromatic groups were observed, while noticeable peak shifts and reduced intensities appeared in the TE-CuONPs spectrum. These spectral changes indicate that phenolics, flavonoids, and organic acids acted as reducing, capping, and surface-functionalizing agents during nanoparticle synthesis. Our findings closely match previously published FTIR profiles of plant-mediated copper nanoparticles, where similar functional groups were implicated in nanoparticle stabilization and coating. The strong negative zeta potential (− 45.8 mV), low PDI (~ 0.22), and uniform nanosized range further support efficient surface functionalization by these phytochemicals, contributing to excellent colloidal stability and enhanced antimicrobial activity. Overall, the comparative FTIR data and physicochemical properties collectively demonstrate that *T. vulgaris* metabolites play a critical role in the successful synthesis, stabilization, and bioactivity of CuO nanoparticles (Ramos-Zúñiga et al. 2023; Mirzaei et al. 2021a; Somaghian et al. 2024; Almoneef et al. 2024).

XRD analysis revealed a crystalline structure with distinct diffraction peaks. The most intense peak at 2θ = 36.5° corresponds to the (111) plane of monoclinic CuO, confirming successful formation of CuO nanoparticles. Minor peaks at lower angles may be attributed to residual phytochemicals or secondary crystalline phases. The sharp diffraction peaks indicate the efficacy of *T. vulgaris* extract as a reducing agent, stabilizing CuO nanoparticles into a distinct crystalline form with high crystallinity. This is comparable to studies using *Azadirachta indica* and *Moringa oleifera* ^46,47^, contrasting with the less defined peaks often seen in non-green synthesis methods (Abd-Elhalim et al. 2019).

SEM–EDX analysis showed that TE-CuONPs predominantly formed nanoscale clusters with relatively uniform distribution, characteristic of biosynthesized nanoparticles with strong inter-particle interactions. EDX confirmed the elemental composition with copper (18.9 wt%), oxygen (64.2 wt%), and minor carbon traces (11.3 wt%) from phytochemicals, consistent with other plant-mediated synthesis studies (Murugan 2018). TEM imaging confirmed predominantly spherical morphology with particle sizes ranging from 15 to 70 nm and a narrow size distribution peaking around 40–50 nm, suggesting controlled synthesis with minimal agglomeration, in agreement with studies on nanoparticles from *Curcuma longa* and *Mentha piperita* (Rajesh et al. 2018).

DLS measurements revealed that TE-CuONPs had a Z-average hydrodynamic diameter of 119.2 nm with excellent monodispersity (PDI = 0.22) and a strong negative zeta potential of − 45.8 mV, indicating good colloidal stability. In contrast, the TE extract showed larger aggregates (Z-average = 990 nm) with a positive zeta potential (+ 13.6 mV), reflecting reduced stability and aggregation tendency. These findings align with previous studies on *Hibiscus rosa-sinensis*-mediated copper nanoparticles (Fouda et al. 2020).

TE-CuONPs exert multimechanistic biological effects through: (1) Cu^2^⁺/Cu⁺ redox cycling catalyzing ROS generation (•OH, O₂•⁻, H₂O₂), causing membrane lipid peroxidation, protein oxidation, and DNA fragmentation; (2) electrostatic interaction of Cu^2^⁺ with negatively charged bacterial membranes, disrupting integrity; (3) intracellular interference with DNA replication, protein synthesis, and ATP production; (4) enhanced cellular uptake via nanoscale dimensions; (5) synergistic effects from *T. vulgaris* phytochemicals (thymol, carvacrol) contributing membrane disruption and stabilization. The green synthesis of copper oxide nanoparticles (CuONPs) using Thymus vulgaris extract involves complex biochemical interactions where the plant’s diverse metabolites, such as polyphenols, flavonoids, and organic acids, play critical roles in both reducing copper ions and stabilizing the formed nanoparticles. These bioactive compounds act as electron donors, facilitating the conversion of Cu(II) ions to CuO nanoparticles through redox reactions while simultaneously capping the nanoparticle surfaces to prevent aggregation and control growth, thus influencing particle size and morphology. The chemical groups present in these metabolites, including hydroxyl, carboxyl, and carbonyl moieties, strongly interact with nanoparticle surfaces, which not only enhances colloidal stability, often indicated by a significant negative zeta potential, but also imparts functional properties such as antioxidant and antimicrobial activities. This surface functionalization facilitates targeted interactions with microbial membranes and cancer cells, promoting enhanced bioactivity by mechanisms such as reactive oxygen species (ROS) production and membrane disruption. Studies underscore the importance of optimizing phytochemical composition and synthesis conditions to tailor nanoparticle characteristics that maximize their therapeutic potential, making the metabolite-nanoparticle interface a focal point for advancing plant-mediated nanomedicine platforms. This nuanced understanding of the molecular mechanisms highlights how the intrinsic properties of the plant extract determine the efficacy, stability, and multifunctionality of the resulting CuONPs in biomedical applications (Marzban et al. 2022; Shakib et al. 2023; Mirzaei et al. 2021b).

### In vitro biological activities

#### Antimicrobial efficacy

**Fig. 7:**
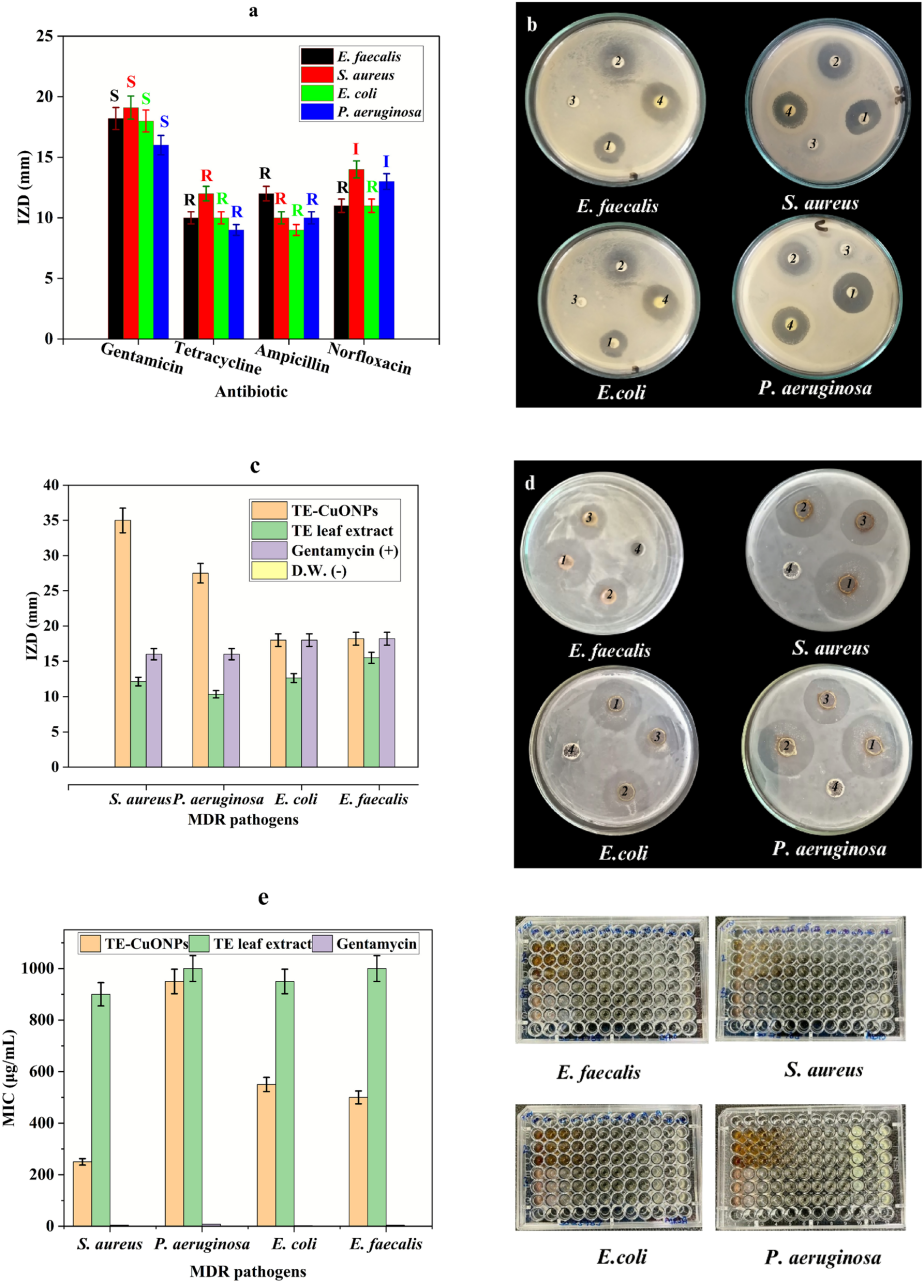

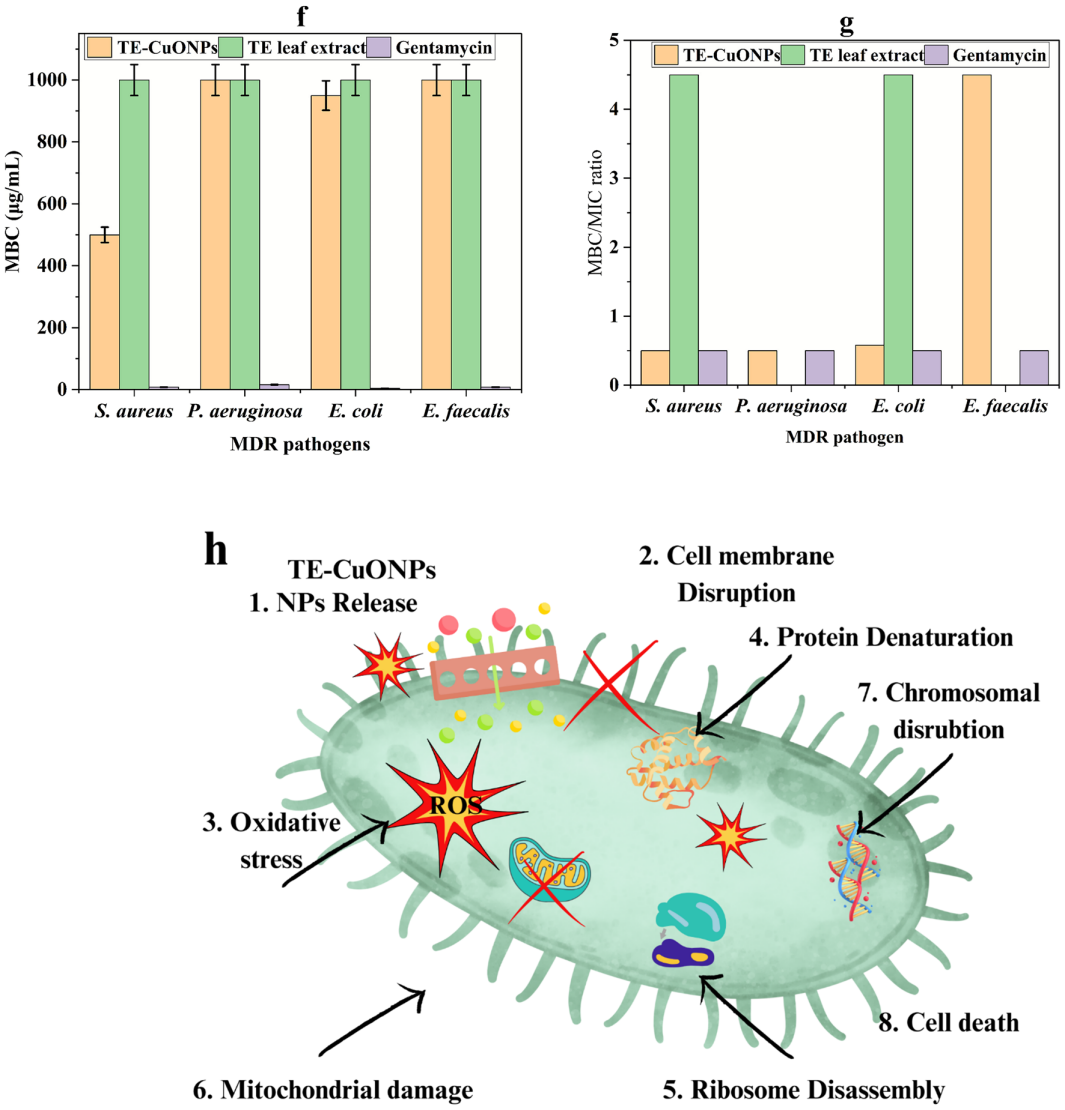
Antimicrobial susceptibility profiles and efficacy of TE-CuONPs against MDR bacterial pathogens. **a** Inhibition zone diameters for four MDR bacterial strains against conventional antibiotics **b**-**e** Disk diffusion assays for *E. faecalis*, *S. aureus*, *E. coli*, and *P. aeruginosa* (disk positions: 1 = tetracycline, 2 = ampicillin, 3 = norfloxacin, 4 = gentamicin), confirming MDR phenotypes with gentamicin susceptibility only. **f** Comparative analysis showing TE-CuONPs activity (17.5–18.5 mm) significantly exceeds TE alone (5.5–8.5 mm, *p* < 0.05) and approaches gentamicin efficacy (20.0–25.0 mm). **g** MIC and MBC values demonstrating bactericidal activity of TE-CuONPs (MBC/MIC ≤ 0.58) against *S. aureus*, *P. aeruginosa*, and *E. coli*, with bacteriostatic response for *E. faecalis* (MBC/MIC > 1.05). **h** Schematic proposal for antimicrobial mechanisms. Different letters indicate statistical significance (*p* < 0.05, ANOVA/Tukey). TE-CuONPs exhibit multimechanistic antibacterial activity through ROS generation, membrane disruption, and cellular interference

Antimicrobial susceptibility (Fig. 7) testing revealed that all bacterial isolates exhibited multidrug resistance (MDR) profiles, with gentamicin demonstrating the highest efficacy across all strains (*S. aureus*: 25.00 ± 2.45 mm; *P. aeruginosa*: 23.00 ± 2.00 mm; *E. faecalis*: 22.00 ± 2.16 mm; *E. coli*: 20.00 ± 1.83 mm). The activity of gentamicin is attributed to its binding to the 30S ribosomal subunit, causing irreversible inhibition of protein synthesis (Bennett et al. 1988). Although the genetic basis of resistance was not tested in the current study, bacterial resistance to antibiotics is usually mediated by specific genes, individually or combined. Resistance to ampicillin was observed across all isolates. This could be primarily due to β-lactamase production, particularly ESBLs in *E. coli* and intrinsic *Amp*C expression in *P. aeruginosa*, coupled with reduced outer membrane permeability and efflux pump activity (Torrens et al. 2019; Dehbashi et al. 2018). Tetracycline resistance is mediated by active efflux systems (*tet* genes) and ribosomal protection proteins Connell et al. (2003) while fluoroquinolone resistance usually results from chromosomal mutations in *gyrA* and *parC* genes, plasmid-mediated resistance genes (*qnr*, *aac(6')-Ib-cr*), and efflux pumps such as MexAB-OprM in *P. aeruginosa* (Kherroubi et al. 2024; Shariati et al. 2022). The retained gentamicin susceptibility was explained by the absence of aminoglycoside-modifying enzymes (AMEs) and 16S rRNA methyltransferases (Yeganeh Sefidan et al. 2019; Wachino et al. 2020).

The antimicrobial efficacy of TE-CuONPs was evaluated against the same four MDR pathogenic bacteria, demonstrating broad-spectrum bactericidal activity. TE-CuONPs exhibited inhibition zone diameters (IZD) of 17.5–18.5 mm across all strains, significantly exceeding the plant extract alone (5.5–8.5 mm, *p* < 0.05) and approaching gentamicin efficacy (20.0–25.0 mm). Minimum inhibitory concentrations (MICs) ranged from 250 to 950 μg/mL, with minimum bactericidal concentrations (MBCs) of 500–1000 μg/mL, yielding MBC/MIC ratios of 0.50–0.58 for *S. aureus*, *P. aeruginosa*, and *E. coli*, confirming bactericidal activity comparable to gentamicin (ratio: 0.50) according to established criteria (MBC/MIC ≤ 4) (Mirzaei et al. 2023; Nazari et al. 2024). However, *E. faecalis* showed reduced susceptibility (MIC: 950 μg/mL; MBC/MIC > 1.05), indicating bacteriostatic activity, likely due to its thick peptidoglycan layer limiting nanoparticle penetration (Skrzyniarz et al. 2023). Nanoformulation enhanced antimicrobial potency through 2.2- to 3.3-fold increases in IZD and up to fourfold reductions in MIC values compared to the crude extract.

The bactericidal activity of TE-CuONPs is attributed to multiple synergistic mechanisms. Copper oxide nanoparticles generate reactive oxygen species (ROS), including hydroxyl radicals, superoxide anions, and hydrogen peroxide, through Cu^2^⁺/Cu⁺ redox cycling, inducing oxidative stress that causes membrane lipid peroxidation, protein oxidation, and DNA fragmentation (Rakshit et al. 2018; Guo et al. 2020). Positively charged Cu^2^⁺ ions interact electrostatically with negatively charged bacterial membranes, disrupting membrane integrity and causing intracellular content leakage (Kumar et al. 2021; Zheng et al. 2026). Additionally, internalized nanoparticles interfere with DNA replication, protein synthesis, and ATP production, while their nanoscale dimensions (< 100 nm) enable enhanced cellular uptake and penetration (Liu et al. 2020; Zhang et al. 2023).

TE-CuONPs showed enhanced activity against *S. aureus* (MIC: 250 μg/mL) compared to chemically synthesized CuO nanoparticles (MIC: 500–1000 μg/mL), suggesting that phytochemical functionalization improves antimicrobial efficacy. Similar results have been reported for green-synthesized metallic nanoparticles using plant extracts, with MIC ranges of 200–800 μg/mL against pathogenic bacteria (Begum et al. 2022; Dubey et al. 2024). The reduced efficacy against *E. faecalis* is consistent with reports of Gram-positive enterococci exhibiting tolerance through antioxidant systems and biofilm formation (Gaca and Lemos 2019; Yang et al. 2024). While gentamicin exhibited higher potency (MIC: 2–8 μg/mL), TE-CuONPs offer several advantages, including multi-target action that reduce the likelihood of resistance development, broad-spectrum bactericidal activity, environmentally sustainable synthesis, and cost-effectiveness (Rai et al. 2017; Vundela et al. 2022).

These findings demonstrate that gentamicin remains effective against MDR pathogens and emphasize the need for antimicrobial stewardship to prevent resistance development (Majumder et al. 2020). TE-CuONPs represent a promising alternative antimicrobial agent with potential clinical applications. Future studies should focus on particle size optimization, surface functionalization to enhance penetration against *E. faecalis*, combination therapy approaches, comprehensive cytotoxicity assessments, and in vivo efficacy evaluation to establish clinical viability (Samiei et al. 2016).

### Antibiofilm activity

**Fig. 8:**
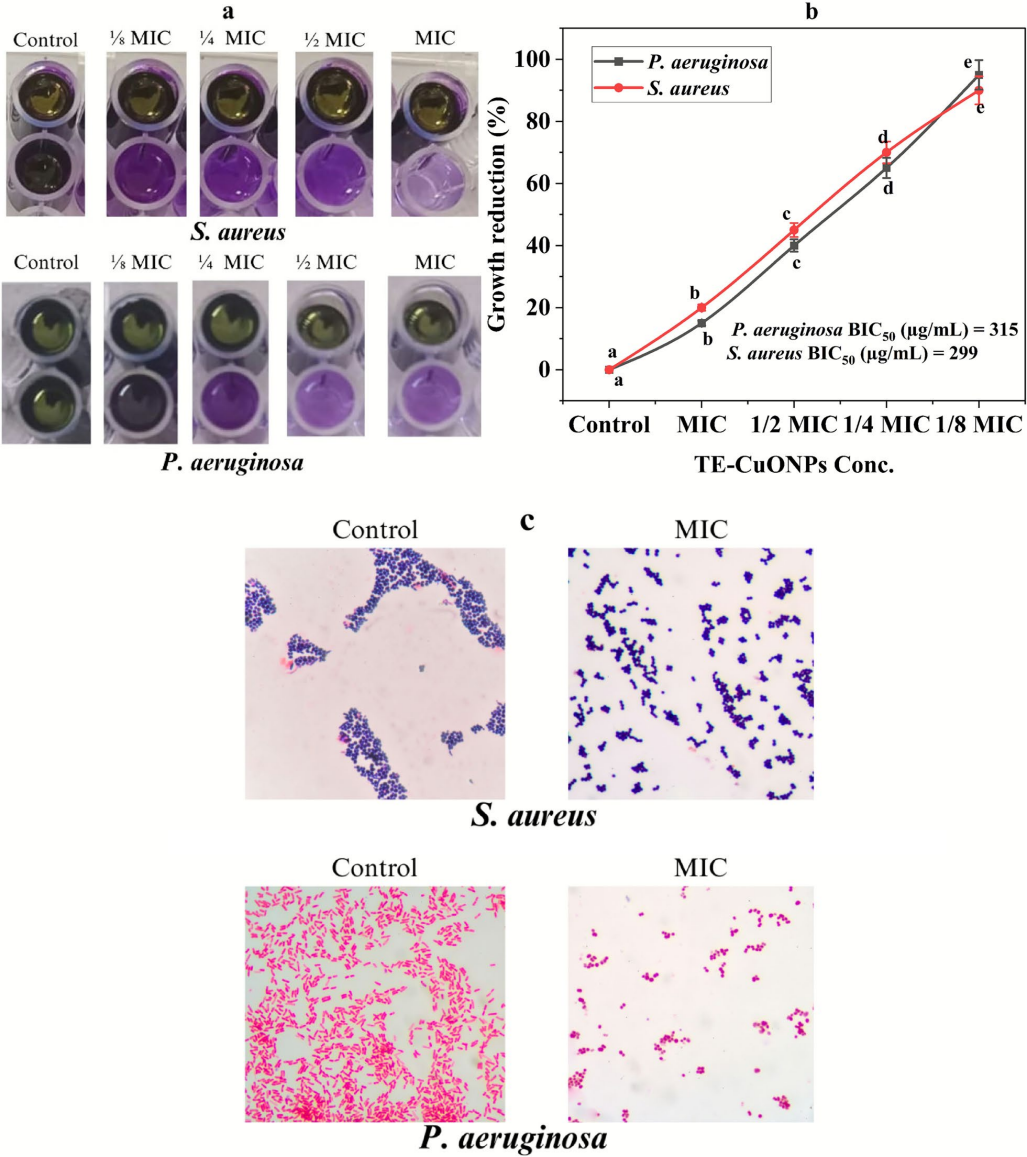
TE-CuONPs exhibit potent antibiofilm activity against *S. aureus* and *P. aeruginosa*. **a** Dose-dependent inhibition of biofilm formation by te-cuonps in *S. aureus* and *P. aeruginosa* assessed by crystal violet assay and spectrophotometric quantification **b** dose–response curves with BIC₅₀ values **c** microscopic visualization (100 ×) demonstrating biofilm disruption at MIC concentration compared to untreated controls

The results in Fig. 8 demonstrate that TE-CuONPs exhibit a dose-dependent growth reduction on both *S. aureus* and *P. aeruginosa*. The percentage growth inhibition increased progressively with decreasing TE-CuONPs concentrations from MIC to 1/8 MIC, with BIC₅₀ values of 315 µg/mL for *P. aeruginosa* and 299 µg/mL for *S. aureus*, indicating slightly higher susceptibility of *S. aureus*. Visual biofilm assays corroborated these findings, showing a marked decrease in biofilm biomass with increasing TE-CuONPs concentrations, as evidenced by the fading of crystal violet staining. At MIC and 1/2 MIC, biofilm formation was substantially impaired compared to the control, with near-complete inhibition at MIC concentration for both strains. These results highlight the potent antibiofilm activity of TE-CuONPs, corroborating their efficacy in disrupting established biofilms and inhibiting bacterial growth within biofilms, suggesting their potential as effective agents for biofilm-related infection control.

### Synergistic antimicrobial activity with gentamicin

#### Checkerboard synergy analysis

**Fig. 9:**
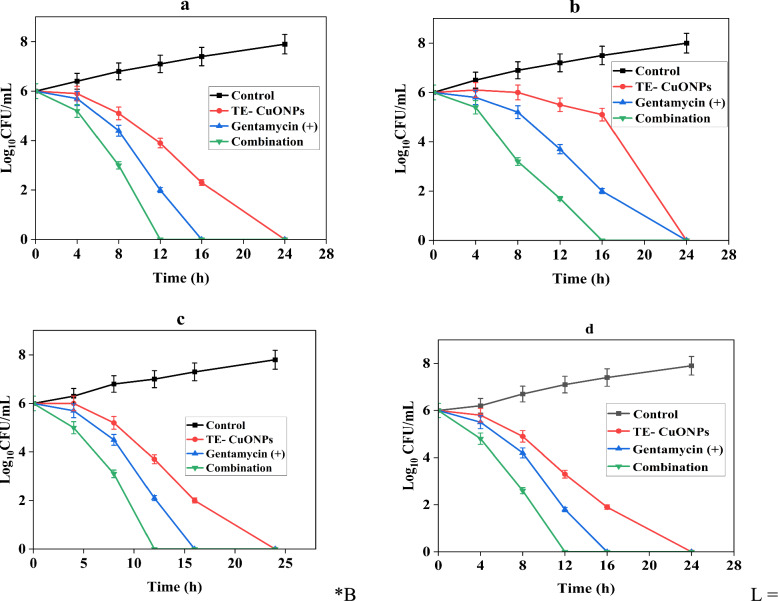
Time-kill kinetics of TE-CuONPs, gentamicin, and combination therapy

Checkerboard assays revealed strong synergy (FICI = 0.13–0.28) with eightfold MIC reductions for both agents against all strains (Table 5). Time-kill kinetics (Fig. 9) showed combination therapy achieved bactericidal endpoints (≥ 3-log₁₀ reduction) 8–12 h faster than monotherapies. This arose from complementary mechanisms: TE-CuONPs disrupted membranes via ROS/lipid peroxidation, enhancing gentamicin penetration and ribosomal binding while overwhelming antioxidant defenses. This multi-target mechanism reduces selection pressure for resistance development compared to single-agent therapy.

**Table 5:** Synergistic antimicrobial activity of TE-CuONPs with gentamicin

Microorganism	MIC Alone (μg/mL)	MIC in Combination (μg/mL)	FIC	FICI	Interpretation
	TE-CuONPs / Gentamicin	TE-CuONPs / Gentamicin	TE-CuONPs / Gentamicin		
*S. aureus*	250 / 4	31.25 / 0.5	0.125 / 0.125	0.25	Synergy
*P. aeruginosa*	500 / 8	62.5 / 1.25	0.125 / 0.156	0.28	Synergy
*E. coli*	550 / 4	68.75 / 0.25	0.125 / 0.0625	0.1875	Synergy
*E. faecalis*	950 / 8	59.38 / 0.50	0.0625 / 0.0625	0.125	Synergy

FICI calculation: FIC_A + FIC_B, where FIC = (MIC in combination)/(MIC alone). Interpretation: ≤ 0.5 = Synergy; > 0.5–1.0 = Additive; > 1.0–4.0 = Indifferent; > 4.0 = Antagonism. Values represent the mean of three independent experiments performed in duplicate (n = 3)

Based on the observed phenotypic resistance patterns, *S. aureus* exhibited *mec*A-mediated β-lactam resistance, *P. aeruginosa* showed MexAB-OprM efflux activity and *Amp*C β-lactamase production, *E. coli* demonstrated extended-spectrum β-lactamase (ESBL) production along with AcrAB-TolC efflux pump activity, while *E. faecalis* possessed intrinsic β-lactamase and efflux-based resistance systems. The multi-targeted antibacterial mechanism of TE-CuONPs enables them to overcome these individual gene-mediated resistance pathways, thereby enhancing their overall antimicrobial efficacy. The pronounced synergy (FICI 0.13–0.28) observed across all tested MDR strains indicates a clinically meaningful reduction in antibiotic dosage. In *S. aureus*, the combination therapy lowered the effective gentamicin concentration from 4 to 0.5 μg/mL, an eightfold decrease, thereby potentially reducing the risk of aminoglycoside-associated nephrotoxicity and ototoxicity. The most potent synergistic effect was noted against *E. faecalis* (FICI = 0.125), the strain exhibiting the highest resistance to TE-CuONPs alone, suggesting that the combination successfully circumvents intrinsic resistance mechanisms such as thick peptidoglycan barriers.

#### Time-kill kinetics and bactericidal enhancement

Time-kill assays (Fig. 9) demonstrated TE-CuONPs achieved complete bacterial eradication within 24h for *S. aureus*, *P. aeruginosa*, and *E. coli*, comparable to gentamicin efficacy. Against *E. faecalis*, TE-CuONPs reduced counts to 2.5 × 10⁷ CFU/mL, showing partial but notable activity despite this organism's enhanced resistance. The crude extract showed minimal activity across all strains (≤ 1-log reduction). The organism-specific susceptibility pattern (*S. aureus* > *P. aeruginosa* ≈ *E. coli* > *E. faecalis*) reflects cell wall structural differences. Critically, the combination achieved clinically defined bactericidal endpoints (≥ 3-log₁₀ reduction from initial inoculum) within 8–12 h, substantially faster than 18–24 h for monotherapies. Statistical analysis using two-way repeated measures ANOVA revealed a significant treatment–time interaction (F = 156.3, *p* < 0.0001). The combination therapy achieved bactericidal endpoints markedly faster than either monotherapy, reaching complete kill of *S. aureus* at 8 h compared to 20 h (*p* < 0.001) and *P. aeruginosa* at 12 h compared to 24 h (*p* < 0.001).

Below detection limit (< 10 CFU/mL or < 1.0 log₁₀ CFU/mL). Test conditions: TE-CuONPs at ½ MIC (125 μg/mL for *S. aureus*, 250 μg/mL for *P. aeruginosa*); gentamicin at ½ MIC (2 μg/mL for *S. aureus*, 4 μg/mL for *P. aeruginosa*). Values represent mean ± standard error from three independent experiments (SE < 0.3 log₁₀ units for all measurements). Combination achieved bactericidal endpoint (≥ 3-log₁₀ reduction) 6–12 h faster than monotherapies.

### In Vitro anticancer activity and apoptotic mechanism

**Fig. 10:**
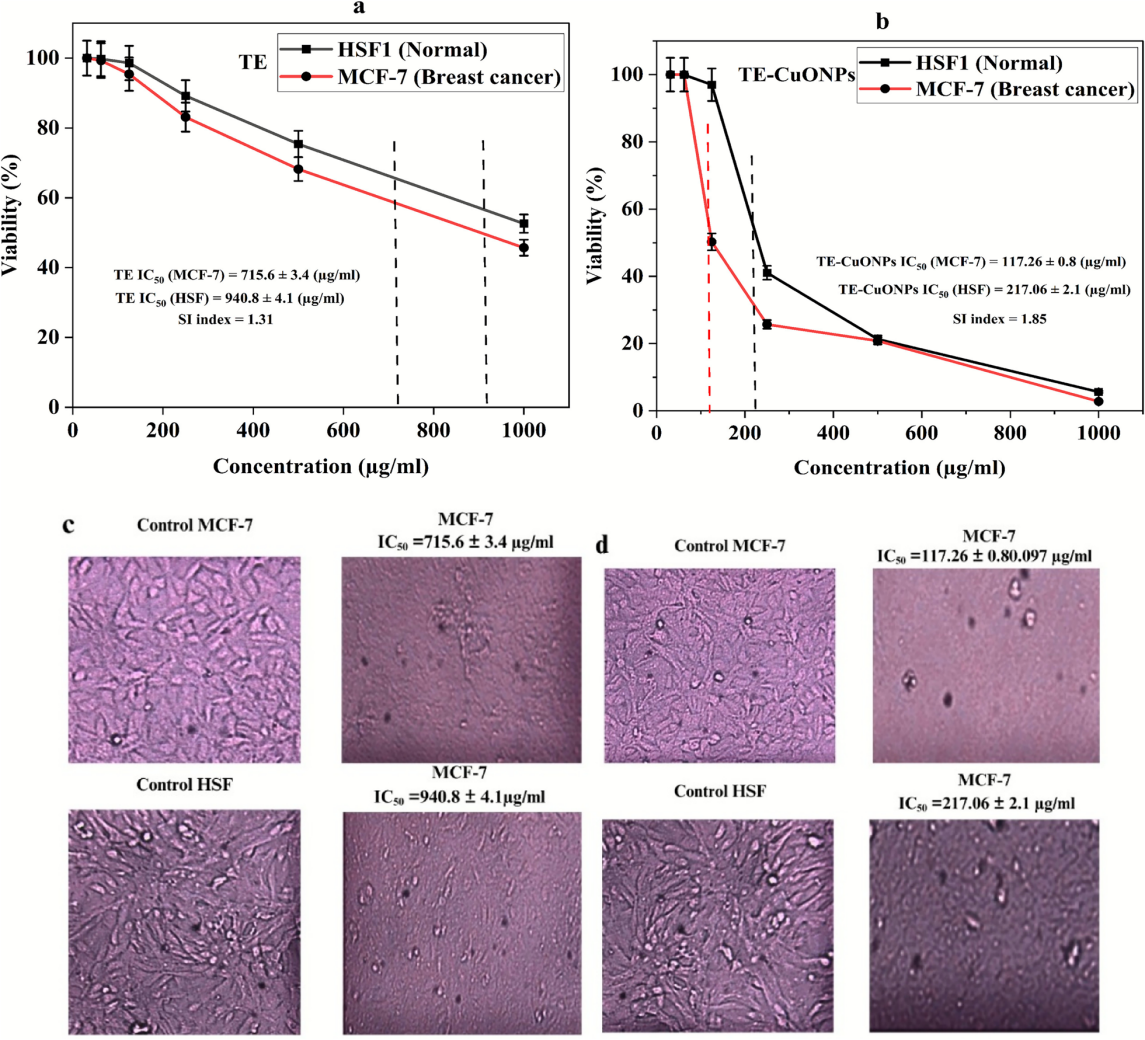

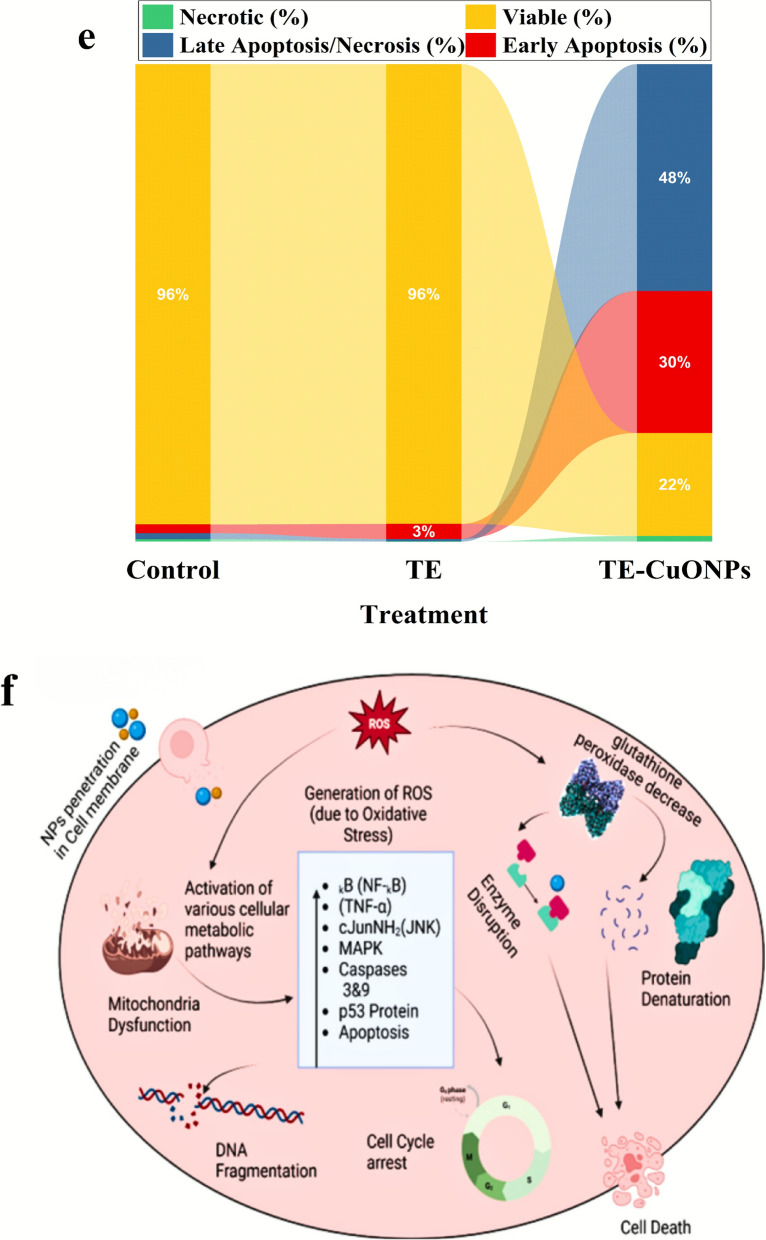

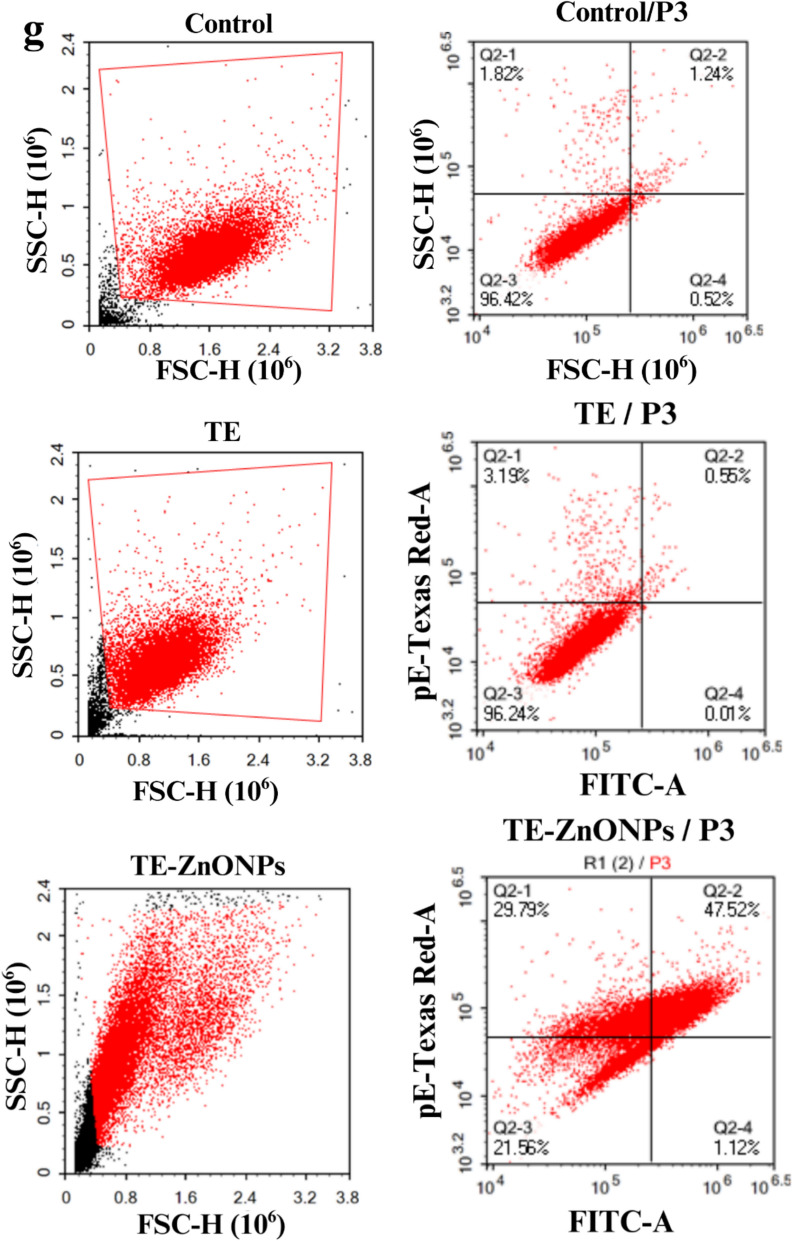
Comprehensive Evaluation of TE-CuONPs Anticancer Activity and Apoptotic Mechanism in MCF-7 Breast Cancer Cells. Concentration-dependent cytotoxicity and mechanistic analysis. **a**, **b** MTT dose–response curves against MCF-7 breast cancer cells and normal human skin fibroblasts (HSF). **c**, **d** Representative phase-contrast micrographs (200 × magnification) of MCF-7 cells after 24 h treatment at respective IC₅₀ concentrations, showing **c** minimal morphological changes with TE treatment versus **d** characteristic apoptotic features with TE-CuONPs treatment, including cell shrinkage (red arrows), membrane blebbing (yellow arrows), chromatin condensation, and cellular detachment. **e** Flow cytometric scatter plots (FSC-H vs. SSC-H) demonstrating consistent cell populations across treatments (10,000 events gated per sample). (**f**) Schematic proposal for anticancer mechanisms. **g** Annexin V-FITC/propidium iodide (PI) dual-staining flow cytometric analysis

TE-CuONPs exhibited concentration-dependent antiproliferative activity against MCF-7 breast cancer cells with IC₅₀ = 117.26 ± 0.80 μg/mL, demonstrating 6.1-fold enhanced potency compared to crude *T. vulgaris* extract (IC₅₀ = 715.6 ± 3.4 μg/mL) and modest cancer cell selectivity (SI = 1.85 vs. normal HSF cells, IC₅₀ = 217.06 ± 2.1 μg/mL) (Fig. 10a, b). Phase-contrast microscopy revealed characteristic apoptotic morphology, including cell shrinkage, membrane blebbing, and detachment in TE-CuONPs-treated cells (Fig. 10c, d). Flow cytometric analysis using Annexin V-FITC/PI dual staining definitively established apoptosis as the primary mechanism of cell death (Fig. 10 e, g). While untreated controls maintained 96.42% viability with 3.06% background apoptosis, and crude extract showed negligible effects (96.24% viable, 3.68% apoptotic at IC₅₀), TE-CuONPs induced pronounced apoptotic cell death with 77.25% total apoptosis (29.73% early, 47.52% late). a 21-fold enhancement. while reducing viability to 21.56%. Critically, primary necrosis remained low (1.19%), indicating regulated programmed cell death rather than non-specific cytotoxicity. The predominance of late-stage apoptosis suggests rapid progression through the apoptotic cascade, consistent with copper-catalyzed ROS generation, mitochondrial dysfunction, and caspase activation. Enhanced activity compared to the crude extract reflects increased cellular uptake of nanoscale particles (40–50 nm), sustained intracellular copper release, and synergistic effects of surface-adsorbed phytochemicals (quercetin 55.92%, chlorogenic acid 15.33%). These findings, combined with demonstrated antimicrobial synergy (FICI = 0.13–0.28), validate TE-CuONPs as a dual-function therapeutic platform warranting preclinical evaluation in animal models.

### DPPH radical scavenging activity and dual functionality

**Fig. 11:**
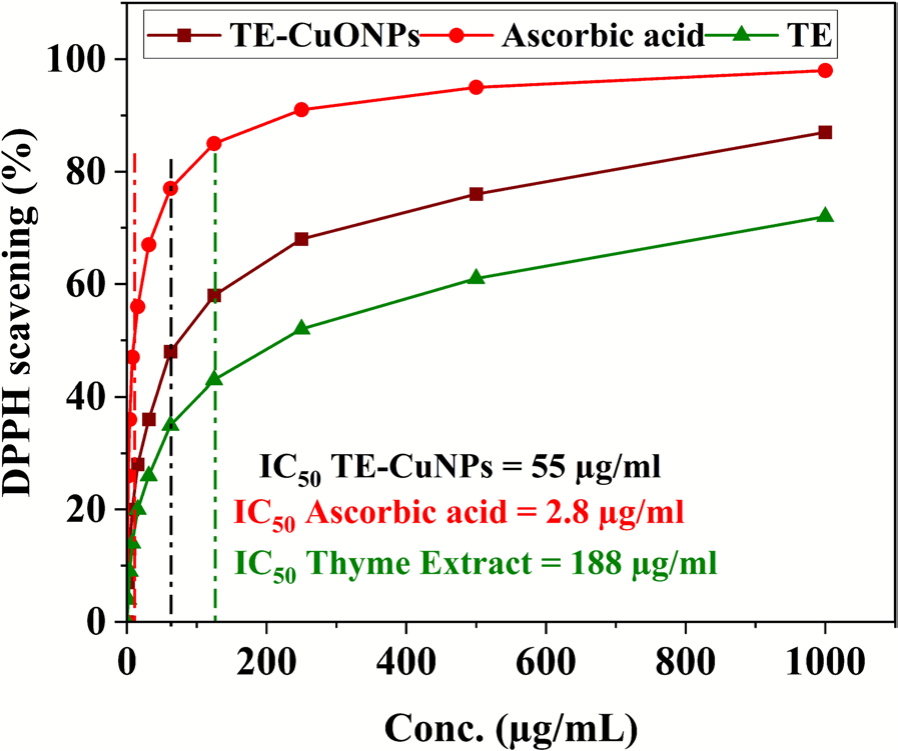
DPPH radical scavenging activity of TE-CuONPs, *T. vulgaris* leaf extract (TE), and ascorbic acid across concentration range (1.95–1000 µg/mL), showing IC₅₀ values and percentage inhibition

TE-CuONPs exhibited concentration-dependent free radical scavenging activity (Fig. 11), achieving 87% DPPH inhibition at 1000 µg/mL with IC₅₀ of 55 µg/mL, representing 3.4-fold enhancement over leaf extract alone (72% inhibition, IC₅₀ = 187.9 µg/mL) (Fig. 10). Ascorbic acid demonstrated superior activity (98% inhibition, IC₅₀ = 2.8 µg/mL), serving as positive control. The improved antioxidant capacity of TE-CuONPs compared to crude extract confirms successful phytochemical incorporation and potential for redox modulation (Chen et al. 2022). The apparent redox paradox observed for TE-CuONPs can be rationalized by their environment- and concentration-dependent behavior, which is well recognized for metal oxide and phytogenic nanomaterials. In the DPPH assay, TE-CuONPs primarily act as direct radical scavengers in a simple, cell-free system, yielding an overall antioxidant readout. In contrast, within cancer cells that already exhibit elevated basal ROS, lysosomal acidification promotes partial CuO dissolution and Cu^2^⁺ release, enabling Fenton-like reactions and amplification of intracellular oxidative stress leading to cytotoxicity. Such switching between antioxidant and pro-oxidant modes with increasing dose and in biologically complex microenvironments has been repeatedly documented for CuO and other transition metal oxide nanoparticles and is considered a key advantage for selective cancer cell killing. These findings align with previous reports showing that CuO nanoparticles potently induce ROS-mediated damage and oxidative stress responses in mammalian cells (Fahmy and Cormier 2009; Murugesan et al. 2025), supporting the plausibility of the proposed mechanism in the present study.

## Conclusion

This study establishes a well-defined green synthesis protocol for *Thymus vulgaris*-mediated copper oxide nanoparticles (TE-CuONPs) optimized using Box-Behnken design, demonstrating how metal precursor concentration, reaction time, and temperature influence nanoparticle yield and optical properties. The synthesized TE-CuONPs exhibited favorable physicochemical characteristics, including narrow size distribution, colloidal stability, and crystalline structure, attributed to phytochemical-mediated reduction and stabilization. In vitro evaluations revealed concentration-dependent antimicrobial activity against multidrug-resistant pathogens, selective cytotoxicity toward MCF-7 breast cancer cells (SI = 1.85) with apoptotic morphological changes, and enhanced antioxidant capacity compared to crude extract in cell-free assays.

### Study limitations and future perspectives

While this study successfully establishes proof-of-concept for the anticancer potential of green-synthesized TE-CuONPs, several limitations warrant acknowledgment. The selectivity index (SI = 1.85) against MCF-7 cells falls marginally below the ideal clinical threshold of SI > 2, and our mechanistic investigation, though providing preliminary evidence of apoptotic induction through morphological assessment and flow cytometry, does not comprehensively elucidate the underlying molecular pathways or systematically quantify the concentration threshold at which TE-CuONPs transition from antioxidant (DPPH scavenging) to pro-oxidant (ROS generation) behavior. Future research should prioritize surface functionalization with targeted ligands (folic acid, RGD peptides) to achieve clinically relevant selectivity, Western blot analysis of key apoptotic regulators (caspase-3/9, Bax/Bcl-2 ratio, p53, cytochrome c), dose-dependent intracellular ROS quantification using fluorescent probes (DCFH-DA, MitoSOX) across therapeutic concentrations (10–1000 µg/mL) in both normal and cancer cells to definitively establish the antioxidant-to-pro-oxidant transition threshold, evaluation of glutathione depletion and lipid peroxidation as oxidative stress markers, and in vivo validation using xenograft tumor models to assess pharmacokinetics, biodistribution, tumor suppression efficacy, and systemic toxicity profiles. Despite these limitations, our findings align with established copper nanoparticle literature demonstrating context-dependent redox behavior and provide a robust foundation for *Thymus vulgaris*-mediated green synthesis of CuONPs as a promising anticancer nanotherapeutic platform for subsequent optimization and translational studies.

## Supplementary Information

Supplementary file 1.

## Data Availability

The original and processed data supporting the findings of this study can be obtained from the corresponding author upon reasonable request.
