# Supplementary material for: Box-Behnken optimized copper oxide nanoparticles from Thymus vulgaris potentiate efficacy against multidrug-resistant bacterial pathogens and exhibit anticancer activity
Source: Bioresour Bioprocess. 2026 Feb 11;13(1):23. doi: 10.1186/s40643-026-01008-5 (PMC12891328; doi:10.1186/s40643-026-01008-5)
Supplement: Supplementary file 1 [file 40643_2026_1008_MOESM1_ESM.docx]

**Box-Behnken optimized copper oxide nanoparticles from *Thymus vulgaris* potentiate efficacy against multidrug-resistant bacterial pathogens and exhibit anticancer activity**

**Samah H. Abu-Hussien^1*^, Akebe Luther King^2,3^, Muhammad A. Khan^4^**

^1^Department of Agricultural Microbiology, Faculty of Agriculture, Ain Shams University, Cairo, 11241, Egypt

^2^Total Environment Research (TEN-R) Group, College of Health Sciences, University of KwaZulu-Natal, South Africa

^3^Environmental Research Foundation, Westville, South Africa

^4^Department of Biological Sciences, Faculty of Sciences, International Islamic University (IIU), Islamabad, Pakistan

Corresponding author*: Samah H. Abu-Hussien ([samah_hashem1@agr.asu.edu.eg](mailto:samah_hashem1@agr.asu.edu.eg)),


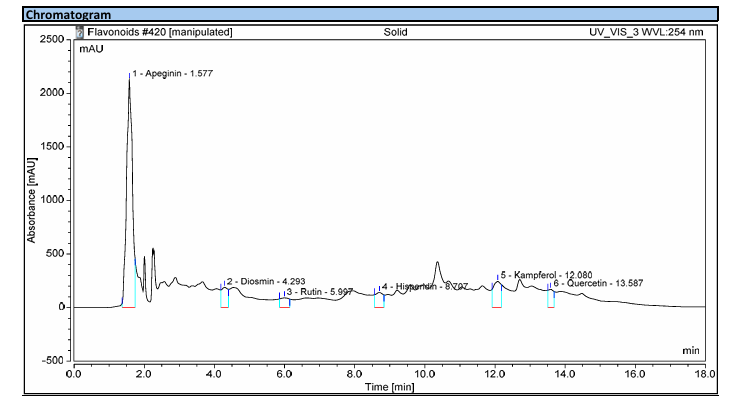


**
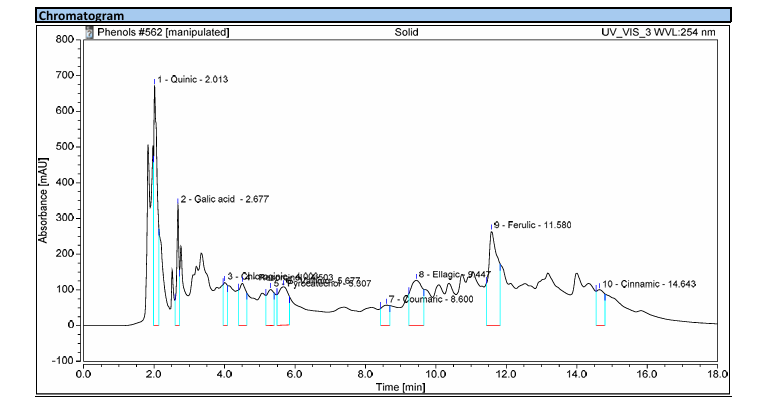
Figure S1: Quantitative profiling of polyphenolics in RJ using HPLC Analysis. A: Flavenoides, b: Phenols**
